# New Bioactive Sesquiterpeniods from the Plant-Derived Endophytic Fungus *Schizophyllum* sp. HM230

**DOI:** 10.3390/jof11040275

**Published:** 2025-04-01

**Authors:** Shi-Yu Li, Lan Yao, Jian-Hua Lv, Zhuang Li, Shuai Xu, Yu Li, Dan Li, Chang-Tian Li

**Affiliations:** 1 Engineering Research Center of Chinese Ministry of Education for Edible and Medicinal Fungi, Jilin Agricultural University, Changchun 130118, China; lishiyu8866@126.com (S.-Y.L.); xushuai@jlau.edu.cn (S.X.); yuli966@126.com (Y.L.); 2 Institute of Biology, Hebei Academy of Science, Shijiazhuang 050000, China; yl52wy@126.com; 3 College of Life Sciences, Hebei Normal University, Shijiazhuang 050000, China; lvjianhua@hebtu.edu.cn (J.-H.L.); lizhuang@hebtu.edu.cn (Z.L.); 4 National-Local Joint Engineering Research Center of Economic Fungus, Jilin Agricultural University, Changchun 130118, China

**Keywords:** *Schizophyllum* sp., sesquiterpenoids, antioxidant activity, antifungal activity

## Abstract

Endophytic fungi provide valuable sources for the discovery of secondary metabolites that can be used as lead compounds in drug discovery. In this study, four new sesquiterpenoids with a farnesane backbone, schizophyllol A–B (**1**–**2**) and schizophylloside A–B (**3**–**4**), together with five known analogues (**5**–**9**), were isolated from the plant-derived fungus *Schizophyllum* sp. HM230. Their structures were established through extensive spectroscopic analyses including HR-ESI-MS and 1D and 2D NMR. The antioxidant activities of all the isolated compounds (compounds **1**–**9**) were evaluated via hydroxyl radical scavenging, DPPH free radical scavenging, and superoxide anion radical scavenging assays. Compounds **1** and **2** displayed stronger antioxidant capacities than the positive control tert-butylhydroquinone. Furthermore, the antifungal activities of the isolated compounds were evaluated against four phytopathogenic fungi: *Sclerotinia ginseng*, *Rhizoctonia solani*, *Cylindrocarpon destructans*, and *Exserohilum turcicum*. All the test compounds demonstrated inhibitory effects; notably, compound **4** exhibited the strongest activities against the four selected phytopathogenic fungi, with inhibitory rates ranging from 42.3% to 65.4% at 0.2 mg/mL.

## 1. Introduction

Plant endophytes can live inside the tissues and organs of plants at any stage or all stages of a plant’s life cycle and do not cause apparent disease symptoms [1,2]. The intricate relationship between these endophytic fungi and their host plants, as well as other organisms, plays a crucial role in facilitating their adaptation to the various biotic and abiotic selection pressures within their respective ecological niches [3,4,5,6]. Recently, plant endophytic fungi have become prominent sources of secondary metabolites [7,8,9]. Song et al. isolated new skeleton dimers with antitumor activity from the plant endophytic fungus *Trematosphaeria terricola* [10]. New antibacterial ansamycin-type polyketides were isolated from the plant endophytic fungus *Streptomyces* sp. [11]. Chen et al. obtained new cytochalasins from the plant endophytic fungus *Phomopsis* sp., which showed antitumor activity [12].

*Vincetoxicum mongolicum* Maxim., a perennial herbaceous plant belonging to the Asclepiadaceae family, is primarily found in the Inner Mongolia autonomous region, the Ningxia hui autonomous region, and the Xinjiang uygur autonomous regions in China [13]. This plant is rich in crude protein, amino acids, and crude fat so can be used as forage grass [14]. *V. mongolicum* has a long history in traditional Chinese medicine for relieving pain, activating blood circulation, resisting inflammation, relieving cough, and removing phlegm. *V. mongolicum* is often used to kill insects, relieve pain, reduce heat, and prevent diarrhea in humans [15]. C21 steroids are the main chemical constituents of this plant, along with acetophenones and alkaloids [16,17,18]. However, reports are scarce regarding the secondary metabolites of its endophytic fungi.

**Figure 1:**
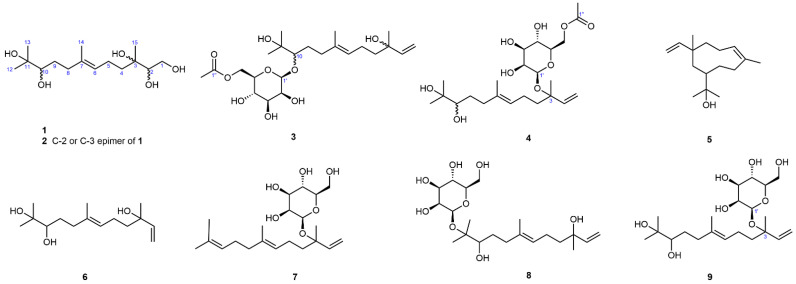
Chemical structures of compounds **1**–**9**.

*Schizophyllum* sp. is widely found in medicinal plants, soil, and marine organisms [19]. The schizophyllan from *S. commune* has been therapeutically applied in antitumor treatment and in drug carriers [20]. Wang et al. reported that the metabolites of *Schizophyllum* sp. from oyster possess acetylcholinesterase-inhibition and antioxidant properties [21]. However, there are few studies on the natural products of the endophytic fungus *Schizophyllum* sp. In our prior study, some alkaloids with antioxidant activity were isolated from the endophytic fungus *Schizophyllum* sp. HM230 of *V. mongolicum* [22]. In addition to these metabolites, several minor secondary metabolites were detected in the solid culture extract during separation. To obtain new natural products, *Schizophyllum* sp. HM230 was refermented at a large scale under the same conditions. Subsequently, the chemical investigation of the EtOAc extract of the fermentation product led to the isolation of four new sesquiterpenoids (**1**–**4**), along with five known analogues (**5**–**9**) (Figure 1). Then, the antioxidant and antifungal activities of isolated compounds were evaluated. This paper provides a description of the isolation, structural elucidation, and biological activities of these compounds.

## 2. Materials and Methods

### 2.1. General Experimental Procedures

Infrared (IR) spectra were recorded on a Thermo Nicolet Avatar FT-IR-750 spectrophotometer using KBr disks (Thermo, Tokyo, Japan). Ultraviolet (UV) spectra were obtained with a TU-1810 UV/vis spectrophotometer (Beijing Persee General Instrument Co., Ltd., Beijing, China). NMR spectra were acquired on a Bruker DRX-600 NMR spectrometer (Bruker, Ettlingen, Germany). HR-ESI-MS data were collected using a Waters Xevo G2 Q-TOF mass spectrometer (Milford, MA, USA). Column chromatography (CC) was conducted using a Sephadex LH-20 (GE Healthcare Biosciences AB, Uppsala, Sweden), silica gel (200–300 mesh, Qingdao Marine Chemical Ltd., Qingdao, China) and YMC RP-18 gel (Fuji Silysia Chemical Ltd., Kasugai, Japan). High-performance liquid chromatography (HPLC) was performed utilizing a Waters 2535 chromatography system (Waters, Milford, MA, USA) that was equipped with a Waters 2489 UV/visible detector and a YMC-Pack ODS-A column (250 × 10 mm, 5 µm) (YMC Co., Ltd., Kyoto, Japan). HPLC-grade solvents, namely acetonitrile and water, were procured from Thermo Fisher Scientific Korea, Ltd. (Seoul, Korea).

### 2.2. Fungal Material and Fermentation

The fungus *Schizophyllum* sp. HM230 was isolated from *V. mongolicum* collected from the Faunas of Alxa Area, Inner Mongolia, in August 2019. The fungus was identified based on its morphological features and ITS sequence data. The ITS sequence was uploaded to GeneBank (No. OP829145). This fungal strain was stored in the Laboratory of Applied Mycology, College of Life Sciences, at Hebei Normal University, China.

The fungus was cultured in four 500 mL Erlenmeyer flasks, each containing 200 mL of Potato Dextrose Broth (potato 200 g and dextrose 20 g per liter), at a temperature of 25 °C on a rotary shaker set at a speed of 120 rpm for a duration of 7 days, with the aim of obtaining the seed culture. Subsequently, the seed culture was introduced into 500 mL Erlenmeyer flasks, each containing solid rice medium. The rice medium was used for solid fermentation, with each 500 mL Erlenmeyer flask containing 100 g of rice and 100 mL of water sterilized at 121 °C for 15 min. The fungal strain was incubated at room temperature for 30 days.

### 2.3. Extraction and Isolation

The total solid fermentation product was extracted in 400 Erlenmeyer flasks using EtOAc three times and concentrated under reduced pressure to obtain the crude extract (57.5 g).

The extract was separated using silica gel CC (1200 g, 200–300 mesh) and eluted with a CH_2_Cl_2_-MeOH gradient system (50:1–1:1) to yield 8 fractions (Fr.1–Fr.8). Fr. 5 (8.1 g) was further separated using silica gel CC (EtOAc-MeOH, 1:0 to 0:1) to produce 7 subfractions (Fr. 5.1–Fr. 5.7). Fr. 5.2 (3.6 g) was subjected to an ODS CC and eluted with mixtures of MeOH and H_2_O (30:70–80:20) to produce 8 subfractions (Fr. 5.2.1–Fr. 5.2.8). Fr.5.2.2 (522 mg) was purified by HPLC (MeCN-H_2_O, 20:80, 2.5 mL/min) to afford **1** (29.0 mg, *t*_R_ = 11.0 min) and **2** (36.0 mg, *t*_R_ = 12.2 min). Fr.5.2.7 was purified by HPLC (MeCN-H_2_O, 28:72, 2.5 mL/min) to afford **3** (23.1 mg, *t*_R_ = 22.1 min) and **4** (18.0 mg, *t*_R_ = 23.6 min). Fr. 6 (7.2 g) was fractionated via silica gel CC (EtOAc-MeOH, 1:0 to 0:1) to produce 8 subfractions (Fr. 6.1–Fr. 6.8). Fr. 6.4 (893 mg) was subjected to a Sephadex LH-20 column and eluted with MeOH to obtain compounds **6** (20.6 mg) and **7** (33.8 mg). Fr. 7 (12.2 g) was separated using ODS CC and eluted with mixtures of MeOH and H_2_O (20:80–80:20) to produce 6 subfractions (Fr. 7.1–Fr. 7.6). Fr. 7.2 (322 mg) was subjected to a Sephadex LH-20 column and eluted with MeOH to obtain compound **8** (40.2 mg). Compounds **5** (13.7 mg, *t*_R_ = 21.5 min) and **9** (19.1 mg, *t*_R_ = 24.8 min) were obtained from Fr. 7.4 (80 mg) via HPLC elution with CH_3_CN and water (20:80).

Schizophyllol A (**1**) was obtained as a white amorphous powder. UV (MeOH) *λ*max (log *ε*) 200 (4.04) nm; IR (KBr) *v*_max_ cm^−1^: 3341.90, 1379.73, 1074.87, 1026.54; ^1^H and ^13^C NMR data, see Table 1 and Table 2; HR-ESI-MS *m*/*z* 313.1973 [M+Na]^+^ (calcd for C_15_H_30_O_5_).

Schizophyllol B (**2**) was obtained as a white amorphous powder. UV (MeOH) *λ*max (log *ε*) 200 (4.75) nm; IR (KBr) *v*_max_ cm^−1^: 3341.33, 1381.33, 1079.54, 1027.11. ^1^H and ^13^C NMR data, see Table 1 and Table 2; HR-ESI-MS *m*/*z* 313.1977 [M+Na]^+^ (calcd for C_15_H_30_O_5_).

Schizophylloside A (**3**) was obtained as a white amorphous powder. UV (MeOH) *λ*max (log *ε*) 200 (7.19) nm; IR (KBr) *v*_max_ cm^−1^: 3334.65, 1726.06, 1069.04, 1025.45; ^1^H and ^13^C NMR data, see Table 1 and Table 2; HR-ESI-MS *m*/*z* 483.2568 [M+Na]^+^ (calcd for C_23_H_40_O_9_).

Schizophylloside B (**4**) was obtained as a white amorphous powder. UV (MeOH) *λ*max (log *ε*) 200 (5.82) nm; IR (KBr) *v*_max_ cm^−1^: 3363.98, 1736.32, 1066.55, 1026.79; ^1^H and ^13^C NMR data, see Table 1 and Table 2; HR-ESI-MS *m*/*z* 483.2560 [M+Na]^+^ (calcd for C_23_H_40_O_9_).

Neroplomacrol (**5**): ^1^H-NMR (CD_3_OD, 600 MHz) *δ*_H_: 5.93 (1H, dd, *J* = 17.4, 10.8 Hz, H-2), 5.18 (1H, m, H-6), 5.18 (1H, m, H-1a), 5.05 (1H, dd, *J* = 10.8, 1.5 Hz, H-1b), 3.25 (1H, dd, *J* = 10.5, 1.7 Hz, H-10), 1.64 (3H, s, H-14), 1.27 (3H, s, H-15), 1.15 (3H, s, H-12), 1.18 (3H, s, H-13); ^13^C-NMR (CD_3_OD, 150 MHz) *δ*_C_: 146.4 (C-2), 136.0 (C-7), 125.8 (C-6), 112.0 (C-1), 79.0 (C-10), 73.9 (C-11), 73.8 (C-3), 43.4 (C-4), 37.8 (C-8), 30.8 (C-9), 27.6 (C-15), 25.6 (C-12), 25.0 (C-13), 23.7 (C-5), 16.1 (C-14).

3,7,11-trimethyl-1,6-dodecadien-3,10,11-triol (**6**): ^1^H-NMR (CD_3_OD, 600 MHz) *δ*_H_: 5.93 (1H, dd, *J* = 17.4, 10.8 Hz, H-2), 5.21 (1H, dd, *J* = 10.8, 1.5 Hz, H-1a), 5.21 (1H, m, overlapped, H-6), 5.05 (1H, dd, *J* = 10.8, 1.5 Hz, H-1b), 3.25 (1H, dd, *J* = 10.6, 1.7 Hz, H-10), 1.64 (3H, s, H-14), 1.27 (3H, s, H-15), 1.18 (3H, s, H-13), 1.15 (3H, s, H-12); ^13^C-NMR (CD_3_OD, 150 MHz) *δ*_C_: 112.0 (C-1), 146.4 (C-2), 73.7 (C-3), 43.4 (C-4), 23.7 (C-5), 125.8 (C-6), 136.0 (C-7), 30.8 (C-8), 37.8 (C-9), 79.0 (C-10), 73.8 (C-11), 25.6 (C-12), 27.6 (C-13), 16.1 (C-14), 25.0 (C-15).

Mannonerolidol (**7**): ^1^H-NMR (CD_3_OD, 600 MHz) *δ*_H_: 5.17 (1H, dd, *J* = 11.0, 1.5 Hz, H-1a), 5.23 (1H, dd, *J* = 17.3, 1.5 Hz, H-1b), 6.02 (1H, dd, *J* = 17.3, 11.0 Hz, H-2), 1.33 (3H, s, 3-CH_3_), 1.64 (2H, t, *J* = 8.5 Hz, H-4), 2.07 (2H, m, H-5), 5.11 (1H, m, H-6), 1.60 (6H, s, 7,11-CH_3_), 1.97 (2H, t, *J* = 7.5 Hz, H-8), 2.04 (2H, m, H-9), 5.08 (1H, m, H-10), 1.67 (3H, s, H-12), 4.59 (1H, d, *J* = 1.0 Hz, H-1′), 3.72 (1H, dd, *J* = 3.3, 1.0 Hz, H-2′), 3.43 (1H, dd, *J* = 9.3, 3.3 Hz, H-3′), 3.57 (1H, dd, *J* = 9.3, 9.3 Hz, H-4′), 3.11 (1H, m, H-5′), 3.80 (1H, dd, *J* = 11.7, 2.5 Hz, H-6′a), 3.69 (1H, dd, *J* = 11.7, 5.5 Hz, H-6′b); ^13^C-NMR (CD_3_OD, 150 MHz) *δ*_C_: 115.0 (C-1), 144.4 (C-2), 81.3 (C-3), 23.9 (C-4), 27.9 (C-5), 125.8 (C-6), 136.0 (C-7), 16.3 (7-CH_3_), 40.8 (C-8), 23.7 (C-9), 125.6 (C-10), 132.2 (C-11), 17.9 (7-CH_3_), 26.0 (C-12), 96.7 (C-1′), 74.3 (C-2′), 75.7 (C-3′), 68.4 (C-4′), 77.9 (C-5′), 62.9 (C-6′).

Icariside C_2_ (**8**): ^1^H-NMR (CD_3_OD, 600 MHz) *δ*_H_: 5.20 (1H, m, overlapped, H-1α), 5.18 (1H, m, overlapped, H-1β), 5.90 (1H, dd, *J* = 17.4, 10.8 Hz, H-2), 4.66 (1H, s, Anomeric), 1.16 (3H, s, H-12), 1.15 (3H, s, H-13), 1.60 (3H, s, H-14), 1.24 (3H, s, H-15); ^13^C-NMR (CD_3_OD, 150 MHz) *δ*_C_: 112.0 (C-1), 146.3 (C-2), 72.5 (C-3), 43.5 (C-4), 23.7 (C-5), 125.6 (C-6), 136.5 (C-7), 30.8 (C-8), 37.3 (C-9), 75.5 (C-10), 78.0 (C-11), 24.8 (C-12), 27.6 (C-13), 16.2 (C-14), 26.8 (C-15), 103.2 (C-1′), 74.5 (C-2′), 87.9 (C-3′), 68.4 (C-4′), 73.9 (C-5′), 62.8 (C-6′).

Icariside C_3_ (**9**): ^1^H-NMR (CD_3_OD, 600 MHz) *δ*_H_: 5.23 (1H, m, overlapped, H-1α), 5.16 (1H, m, overlapped, H-1β), 6.02 (1H, dd, *J* = 17.7, 10.9 Hz, H-2), 4.60 (1H, s, Anomeric), 1.18 (3H, s, H-12), 1.16 (3H, s, H-13), 1.62 (3H, s, H-14), 1.33 (3H, s, H-15); ^13^C-NMR (CD_3_OD, 150 MHz) *δ*_C_: 115.0 (C-1), 144.2 (C-2), 81.2 (C-3), 41.2 (C-4), 23.6 (C-5), 125.8 (C-6), 136.1 (C-7), 30.7 (C-8), 37.8 (C-9), 79.0 (C-10), 73.8 (C-11), 25.6 (C-12), 23.8 (C-13), 16.2 (C-14), 25.0 (C-15), 96.5 (C-1′), 75.5 (C-2′), 77.9 (C-3′), 68.4 (C-4′), 75.5 (C-5′), 62.9 (C-6′).

### 2.4. Antioxidant Activity Assays

#### 2.4.1. DPPH Radical Scavenging Activity

The 1,1-diphenyl-2-picryl-hydazyl (DPPH) scavenging assay was performed in line with a previously reported method [23]. We mixed 100 μL of DPPH solution (dissolved in methanol at 0.2 mM) with 100 μL of sample at different concentrations. *tert*-Butylhydroquinone (TBHQ) served as the positive control in the experiment. A blank control was prepared by combining 100 μL of sample with 100 μL of methanol, and the negative control was prepared as DPPH solution in methanol. Following a reaction period of 40 min at room temperature under dark conditions, the absorbance of the mixture was quantified at a wavelength of 517 nm using a microplate reader (Thermo, Multiskan FC, Waltham, MA, USA). Each group was repeated thrice. The DPPH radical scavenging rate was calculated as follows:

DPPH scavenging rate (%) = [A_0_ − (A_1_ − A_2_)]/A_0_ × 100

where A_0_ represents the absorbance value of the negative control, A_1_ denotes the absorbance value of the reaction mixture, and A_2_ is the absorbance value of the blank control.

#### 2.4.2. Superoxide Anion Radical Scavenging Assay

The superoxide anion radical scavenging assay was conducted using the previously reported method [24]. The 100 μL samples with different concentrations were added to a 96-well plate, followed by the addition of 100 μL of Tris-HCl buffer (50 mmol/L). The plate was then incubated at 25 °C for 20 min. Subsequently, 7 μL of pyrogallol solution (30 mmol/L) was added and the reaction was allowed to proceed at 25 °C for 6 min. Finally, 7 μL of 0.1 mol/L HCl was added to terminate the reaction. The absorbance at 320 nm was measured and recorded as A_1_. Pyrogallol solution was replaced with 0.1 mol/L HCl and absorbance was measured at 320 nm and recorded as A_2_. The sample solution was replaced with distilled water and absorbance was measured at 320 nm and noted as A_0_. TBHQ served as the positive control in the experiment. Three parallel experiments were conducted for each group.

The superoxide scavenging percentage was calculated as follows:

Scavenging rate (%) = [A_0_ − (A_1_ − A_2_)]/A_0_ × 100

#### 2.4.3. Hydroxyl Radical Scavenging Activity

The determination of the hydroxyl radical scavenging ability was conducted according to previously described methods [25]. The 100 μL samples with different concentrations were added to a 96-well plate, followed by the addition of 30 μL of sodium salicylate solution (20 mmol/L), 100 μL of FeSO_4_ solution (1.5 mmol/L), and 70 μL of H_2_O_2_ solution. The plate was then incubated at 37 °C for 1 h. The absorbance at 510 nm was measured and recorded as A_1_. Sodium salicylate solution was replaced with distilled water and the absorbance was measured at 510 nm and recorded as A_2_. The sample solution was replaced with distilled water and the absorbance was measured at 510 nm and recorded as A_0_. TBHQ served as the positive control in the experiment. Three parallel experiments were conducted for each group.

The hydroxyl radical scavenging percentage was calculated as follows:

Scavenging rate (%) = [A_0_ − (A_1_ − A_2_)]/A_0_ × 100

### 2.5. Antifungal Assay

The phytopathogenic fungi tested in this study were *Sclerotinia ginseng*, *Rhizoctonia solani*, *Cylindrocarpon destructans*, and *Exserohilum turcicum*, which were obtained from the College of Life Sciences, Hebei Normal University, Shijiazhuang, China. Potato dextrose agar (PDA) was used to culture the strains for seven days at 28 °C after retrieval from the storage tube.

The antifungal activities of the compounds were tested using a mycelium growth inhibition method, as previously described [26]. The compounds were dissolved in DMSO and mixed with PDA culture media to a final concentration of 0.2 mg/mL. The media (6 mL) containing pure compounds were then poured into sterile Petri plates (6 cm). A 5 mm mycelial disk of the test pathogens was cut from the 4-day culture and placed at the center of the plate. The mycelial disks on the PDA containing no test compound served as a negative control, and carbendazim (0.2 mg/mL) was used as a positive control. Each treatment was repeated three times and incubated in the dark at 28 °C. Colony diameters were measured after 72 h of incubation. The mycelial growth inhibition rate was calculated according to the following formula:

Growth inhibition rate (%) = [(D_c_ − D_t_)/(D_c_ − D_i_)] × 100

where D_c_ represents the colony diameter of the control group, D_t_ represents the colony diameter of the treated group, and D_i_ represents the initial colony diameter.

## 3. Results and Discussion

### 3.1. Structure Elucidation

**Figure 2:**
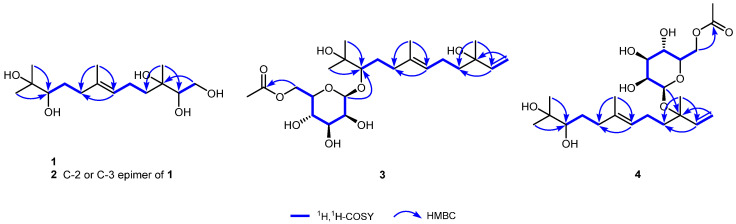
The ^1^H,^1^H-COSY and significant HMBC correlations of compounds **1**–**4**.

Compound **1**, obtained as a white amorphous powder, had a molecular formula of C_15_H_30_O_5_, as determined by the positive HR-ESI-MS (*m*/*z* 313.1973 C_15_H_30_NaO_5_^+^, calcd. 313.1985), indicating one degree of unsaturation. The ^1^H NMR spectrum (Table 1) in CD_3_OD of **1** exhibited one olefinic triplet at *δ*_H_ 5.20 (1H, br t, *J* = 7.0 Hz, H-6), one oxygen-bearing methylene at *δ*_H_ 3.77 (1H, dd, *J* = 11.1, 3.3 Hz, H-1a) and one at 3.53 (1H, dd, *J* = 11.1, 7.9 Hz, H-1b), two oxygen-bearing methines at *δ*_H_ 3.46 (1H, dd, *J* = 7.9, 3.3 Hz, H-2) and 3.23 (1H, dd, *J* = 10.6, 1.5 Hz, H-10), one olefinic methyl signal at *δ*_H_ 1.64 (3H, br s, H-14), and three methyl singlets in the high-field region. The ^13^C NMR spectrum (Table 2) showed a total of fifteen carbon resonances, including two olefinic carbons at *δ*_C_ 136.0 (s, C-7) and 126.0 (d, C-6) due to a trisubstituted double bond group, two oxygen-bearing methine carbons at *δ*_C_ 79.0 (d, C-10) and 78.2 (d, C-2), two oxygen-bearing quaternary carbons at *δ*_C_ 74.9 (s, C-3) and 73.8 (s, C-11), one oxygen-bearing methylene carbon at *δ*_C_ 64.0 (t, C-1), and eight high-field aliphatic carbons (4 × CH_2_ and 4 × CH_3_). The aforementioned NMR features and the degree of unsaturation suggested that **1** is a highly oxidized noncyclic sesquiterpene, structurally related to amarantholidols A–D [27]. The comparison of the NMR data of **1** with those of amarantholidols revealed that their structural differences were due to different oxidation degrees and different oxidation sites. The ^1^H,^1^H-COSY correlations (Figure 2) revealed the existence of three sets of coupling systems. The HMBC correlations (Figure 2) from H-15 [*δ*_H_ 1.13 (3H, s)] to C-2 [*δ*_C_ 78.2 (d)] and C-4 [*δ*_C_ 40.1 (t)], and from H_2_-1 to C-3 [*δ*_C_ 74.9 (s)], revealed that the head isoprenoid was 1,2,3-trihydroxylated. The correlations from H-12 [*δ*_H_ 1.15 (3H, s)] and H-13 [*δ*_H_ 1.12 (3H, s)] to C-10 [*δ*_C_ 79.0 (d)] indicated the appearance of 10,11-dihydroxy at the tail isoprenoid. Furthermore, the *E* configuration of the double bond was determined using the NOESY correlations of H-6↔H_2_-8. Therefore, the structure of **1** was established as (*E*)-3,7,11-trimethyldodec-6-ene-1,2,3,10,11-pentaol, as shown in Figure 1, and named schizophyllol A.

Compound **2**, a white amorphous powder, possessed the same molecular formula (C_15_H_30_O_5_) as that of **1**, according to the positive HR-ESI-MS (*m*/*z* 313.1977 C_15_H_30_NaO_5_^+^, calcd. 313.1985). The ^1^H and ^13^C NMR spectra (Table 1 and Table 2) in CD_3_OD of 2 were highly similar to those of **1**, and the discernable differences only came from the head isoprenoid moiety. Further 2D NMR analysis elucidated the same planar structure as **1**, which indicated that they were a pair of epimers at either C-2 or at C-3. Unfortunately, due to the flexibility of the chain structure, the specific configurations of **1** and **2** could not be determined through the NOESY experiment. Finally, the structure of **2** was established and named schizophyllol B.

Compound **3**, a white amorphous powder, possessed a molecular formula of C_23_H_40_O_9_ according to the positive HR-ESI-MS (*m*/*z* 483.2568 C_23_H_40_NaO_9_^+^, calcd. 483.2565). The ^1^H NMR spectrum (Table 1) in CD_3_OD of **3** exhibited a group of monosubstituted vinyl protons at *δ*_H_ 5.90 (1H, dd, *J* = 17.4, 10.8 Hz, H-2), 5.18 (1H, dd, *J* = 17.4, 1.0 Hz, H-1a), and 5.01 (1H, dd, *J* = 10.8, 1.0 Hz, H-1b); one olefinic triplet at *δ*_H_ 5.16 (1H, br t, *J* = 7.0 Hz, H-6); one sugar anomeric proton at *δ*_H_ 4.60 (1H, br s, H-1′); seven oxygen-bearing protons; one acetoxy methyl singlet; one olefinic methyl broad singlet; and three high-field methyl singlets. The ^13^C NMR spectrum (Table 2) showed a total of twenty-three carbon signals, including one acetoxy carbonyl carbon at *δ*_C_ 172.8 (s); four olefinic carbons at *δ*_C_ 146.3 (d, C-2), 136.7 (s, C-7), 125.6 (d, C-6) and 112.0 (t, C-1) due to two double bond groups; one sugar anomeric carbon at *δ*_C_ 103.4 (d, C-1′); eight oxygenated carbons (2 × C, 5 × CH, 1 × CH_2_); and nine high-field aliphatic carbons (4 × CH_2_ and 5 × CH_3_). One carbonyl, two double bonds, and one hexose ring occupied all four degrees of unsaturation, indicative of the existence of a noncyclic core in **3**. The above NMR features are similar to those of amarantholidoside IV, a noncyclic sesquiterpene glycoside [28]. The same aglycone structure as that of amarantholidoside IV was obtained through further 2D NMR analysis (Figure 2). As a result, their structural differences came from the pyranose moiety. The careful chair conformation analysis, especially the small coupling constant (*J* = 3.2 Hz) of ^3^*J*_H-2′/H-3′_, indicated that H-2′ was in the equatorial orientation, which revealed the existence of mannopyranose moiety. Comparison of the ^13^C NMR data of the sugar residue with those of analogues revealed that the current mannopyranosyl had a β-configuration [29,30]. The HMBC correlation (Figure 2) from the anomeric proton to C-10 [*δ*_C_ 89.0 (d)] allowed for the β-mannopyranosyloxy to be positioned at C-10. The terminal of the sugar residue was acetylated on the basis of the HMBC correlation from H_2_-6′ to the acetoxy carbonyl carbon. Thus, the structure of **3** was established as shown in Figure 1 and named schizophylloside A.

Compound **4** possessed the same molecular formula (C_23_H_40_O_9_) as that of **3**, according to the positive HR-ESI-MS (*m*/*z* 483.2560 C_23_H_40_NaO_9_^+^, calcd. 483.2565). The ^1^H and ^13^C NMR spectra (Table 1 and Table 2) in CD_3_OD of **4** were also very similar to those of **3**, and the remarkable differences were in the ^13^C NMR chemical shifts of C-3, C-10, and C-1′, which suggested their different glycosidation sites. The same aglycone structure as that of **3** was attained through further 2D NMR analysis (Figure 2). The HMBC correlation (Figure 2) from the anomeric proton at *δ*_H_ 4.58 (1H, br s, H-1′) to C-3 [*δ*_C_ 81.2 (s)] positioned the β-mannopyranosyloxy at C-3. Actually, the diagnostic ^13^C NMR glycosylation shift observed for C-3 (∆_δC_ = +7.3 ppm) could also be used to locate the glycosylation position. It should be noted that the current anomeric carbon signal was significantly shifted to the higher field (∆_δC_ = −6.9 ppm), implying glycosylation with a tertiary alcohol, and the ^13^C NMR shift rule was also reflected in some analogs as expected, such as amarantholidoside V. The resulting structure, especially the unusual β-mannopyranosyl moiety, was further verified via 2D NMR analysis. Therefore, the structure of **4** was established as shown in Figure 1 and named schizophylloside B.

By comparing the NMR spectroscopic data with the data in the literature [31,32,33], the five known compounds (**5**–**9**) were identified as neroplomacrol (**5**), 3,7,11-trimethyl-1,6-dodecadien-3,10,11-triol (**6**), mannonerolidol (**7**), icariside C_2_ (**8**), and icariside C_3_ (**9**).

To the best of our knowledge, there is currently no research on the secondary metabolites of the genus of *Schizophyllum*. In this study, we have isolated nine sesquiterpenoid compounds from *Schizophyllum* sp. HM230 for the first time. Compounds **1**–**4** are new compounds, whereas compounds **5**–**9** are known compounds. Our newly identified metabolites from *S. commune* share structural similarities with known sesquiterpene glycosides, such as mannogeranylnerol and mannonerolidol. These compounds are characterized by a glycosylated sesquiterpene aglycone, with mannose as the sugar moiety. The structure of mannogeranylnerol, as described in a recent study [34], exhibits a similar backbone to our compounds, with geranylnerol *β*-D-mannopyranoside as the core structure. Previous studies have demonstrated the antimicrobial bioactivity of mannogeranylnerol and mannonerolidol, suggesting that sesquiterpene glycosides from the *S. commune* may possess antimicrobial properties [32,35]. Accordingly, our compounds may exhibit similar biological activity, warranting further investigation of their pharmacological effects, particularly regarding their potential in fungal pathogenicity and antimicrobial applications. We next tested the antioxidant and antifungal activities of the isolated compounds.

### 3.2. Antioxidant Activities

The degenerative effects caused by the accumulation of free-radical-induced oxidative damage to DNA, proteins, and other macromolecules are major factors that contribute to endogenous damage, leading to aging [36]. Various natural antioxidants can be used to counteract the damage caused by excessive levels of free radicals. The findings are presented in Table 3. In the three antioxidant activity assays, all the tested compounds demonstrated varying levels of antioxidant activity. The results of the hydroxyl radical scavenging assay showed that compounds **1**, **2**, and **6** had stronger scavenging ability, with IC_50_ values of 70.5 ± 3.0, 65.8 ± 2.8, and 80.9 ± 4.1 μM, respectively; these are comparable to those of the TBHQ standard (IC_50_ = 90.8 ± 3.4 μM). Compounds **3**–**5** and **7**–**9** exhibited hydroxyl radical scavenging ability, with IC_50_ values ranging from 170.2 ± 5.2 to 280.6 ± 4.5 μM, respectively. In the DPPH free radical scavenging assays, all the tested compounds exhibited weaker scavenging capacities than the positive control TBHQ. In the superoxide anion radical scavenging assays, compounds **1**–**4** and **9** displayed strong scavenging capacities, with IC_50_ values ranging from 44.6 ± 3.9 to 82.7 ± 3.1 μM, surpassing that of the control (IC_50_ = 140.7 ± 3.2 μM). The superoxide anion radical scavenging capacities of the other compounds were weaker than that of the positive control.

### 3.3. Antifungal Activities

The antifungal activities of compounds **1**–**9** against four phytopathogenic fungi, *S. ginseng*, *R. solani*, *C. destructans*, and *E. turcicum*, were assessed. Carbendazim, a commercially available antifungal agent, was used as a positive control. The results indicated that all the tested compounds had antifungal activities (Table 4). Compound **4** exhibited the highest efficacy against the four chosen phytopathogenic fungi, displaying inhibitory rates ranging from 42.3 to 65.4% at a concentration of 0.2 mg/mL. Compounds **3** and **7**–**9** exhibited moderate antifungal activities, with inhibition rates ranging from 22.5 to 57.1% at the same concentration.

## 4. Conclusions

In summary, four new sesquiterpenoids, schizophyllol A–B (**1**–**2**) and schizophylloside A–B (**3**–**4**), together with five known analogues, were isolated from the plant endophytic fungus *Schizophyllum* sp. HM230 derived from the plant *V. mongolicum*. Their structures were elucidated using various spectroscopic methods. We found that many compounds exhibited antioxidant properties and inhibitory activities against plant pathogens. These findings expand the chemical diversity of sesquiterpeniods and also enrich the secondary metabolites in plant endophytic fungus.

## Figures and Tables

**Table 1:** The ^1^H NMR spectral data for compounds **1**–**4** in CD_3_OD.

No.	1	2	3	4
1	3.53 (1H, dd, 11.1, 7.9) 3.77 (1H, dd, 11.1, 3.3)	3.52 (1H, dd, 11.1, 7.8) 3.72 (1H, dd, 11.1, 3.2)	5.01 (1H, dd, 10.8, 1.0) 5.18 (1H, dd, 17.4, 1.0)	5.15 (1H, dd, 11.0, 1.0) 5.23 (1H, dd, 17.7, 1.0)
2	3.46 (1H, dd, 7.9, 3.3)	3.46 (1H, dd, 7.8, 3.2)	5.90 (1H, dd, 17.4, 10.8)	5.97 (1H, dd, 17.7, 11.0)
4	1.45, 1.58 (each 1H, m)	1.44, 1.58 (each 1H, m)	1.46–1.54 (2H, m)	1.61–1.69 (2H, m)
5	2.04–2.16 (2H, m)	2.09 (2H, q-like, 8.0)	1.95–2.07 (2H, m)	1.99–2.12 (2H, m)
6	5.20 (1H, br t, 7.0)	5.19 (1H, br t, 7.0)	5.16 (1H, br t, 7.0)	5.18 (1H, br t, 7.0)
8	2.01, 2.24 (each 1H, m)	2.01, 2.24 (each 1H, m)	2.04, 2.36 (each 1H, m)	2.00, 2.23 (each 1H, m)
9	1.34, 1.71 (each 1H, m)	1.34, 1.71 (each 1H, m)	1.51, 1.64 (each 1H, m)	1.33, 1.70 (each 1H, m)
10	3.23 (1H, dd, 10.6, 1.5)	3.23 (1H, dd, 10.6, 1.5)	3.37 (1H, overlapped)	3.22 (1H, br d, 10.5)
12	1.15 (3H, s)	1.15 (3H, s)	1.16 (3H, s)	1.15 (3H, s)
13	1.12 (3H, s)	1.12 (3H, s)	1.17 (3H, s)	1.12 (3H, s)
14	1.64 (3H, br s)	1.63 (3H, br s)	1.58 (3H, br s)	1.60 (3H, br s)
15	1.13 (3H, s)	1.14 (3H, s)	1.24 (3H, s)	1.31 (3H, s)
1′			4.60 (1H, br s)	4.58 (1H, br s)
2′			3.98 (1H, br d, 3.2)	3.75 (1H, br d, 3.2)
3′			3.42 (1H, dd, 9.4, 3.2)	3.42 (1H, dd, 9.3, 3.2)
4′			3.53 (1H, t-like, 9.5)	3.50 (1H, t-like, 9.5)
5′			3.37 (1H, overlapped)	3.29 (1H, overlapped)
6′			4.22 (1H, dd, 11.7, 7.0) 4.41 (1H, dd, 11.7, 1.7)	4.17 (1H, dd, 11.7, 7.1) 4.35 (1H, dd, 11.7, 1.8)
2″			2.03 (3H, s)	2.05 (3H, s)

**Table 2:** The ^13^C NMR spectral data for compounds **1**–**4** in CD_3_OD.

No.	1	2	3	4
1	64.0 (t)	64.0 (t)	112.0 (t)	115.0 (t)
2	78.2 (d)	78.5 (d)	146.3 (d)	144.1 (d)
3	74.9 (s)	74.9 (s)	73.9 (s)	81.2 (s)
4	40.1 (t)	39.4 (t)	43.5 (t)	41.0 (t)
5	22.8 (t)	23.1 (t)	23.7 (t)	23.6 (t)
6	126.0 (d)	126.0 (d)	125.6 (d)	125.8 (d)
7	136.0 (s)	136.1 (s)	136.7 (s)	136.1 (s)
8	37.9 (t)	37.9 (t)	37.6 (t)	37.9 (t)
9	30.8 (t)	30.8 (t)	30.8 (t)	30.8 (t)
10	79.0 (d)	79.0 (d)	89.0 (d)	79.0 (d)
11	73.8 (s)	73.8 (s)	74.4 (s)	73.8 (s)
12	25.6 (q)	25.6 (q)	26.5 (q)	25.6 (q)
13	25.0 (q)	25.0 (q)	25.1 (q)	25.0 (q)
14	16.1 (q)	16.1 (q)	16.2 (q)	16.2 (q)
15	22.3 (q)	22.9 (q)	27.6 (q)	24.0 (q)
1′			103.4 (d)	96.5 (d)
2′			72.3 (d)	73.8 (d)
3′			75.2 (d)	75.4 (d)
4′			68.8 (d)	68.8 (d)
5′			75.5 (d)	75.4 (d)
6′			65.4 (t)	65.3 (t)
1′’			172.8 (s)	172.8 (s)
2′’			20.9 (q)	20.9 (q)

**Table 3:** Antioxidative activities of compounds **1**–**9**.

Compounds	IC_50_ (μM)		
	Hydroxyl Radical	Dpph Free Radical	Superoxide Anion Radical
**1**	70.5 ± 3.0	96.8 ± 4.1	55.7 ± 2.5
**2**	65.8 ± 2.8	88.3 ± 5.2	60.2 ± 3.6
**3**	187.5 ± 7.4	141.2 ± 3.2	44.6 ± 3.9
**4**	190.5 ± 6.2	123.8 ± 5.2	77.2 ± 3.1
**5**	216.5 ± 3.2	212.2 ± 3.2	172.6 ± 4.2
**6**	80.9 ± 4.1	102.5 ± 3.6	181.2 ± 3.3
**7**	280.6 ± 4.5	132.2 ± 4.2	162.4 ± 6.1
**8**	170.2 ± 5.2	122.4 ± 2.9	159.5 ± 2.9
**9**	175.5 ± 4.5	126.7 ± 4.2	82.7 ± 3.1
TBHQ ^a^	90.8 ± 3.4	20.2 ± 1.6	140.7 ± 3.2

^a^ TBHQ was used as a positive control. Values are shown as mean ± standard deviation (n = 3).

**Table 4:** Antifungal activity of compounds **1**–**9** at 0.2 mg/mL.

Compound	Inhibition Rate (%)			
	*S. ginseng*	*R. solani*	*C. destructans*	*E. turcicum*
**1**	10.2 ± 2.0	15.5 ± 2.5	6.4 ± 2.2	13.2 ± 1.7
**2**	8.6 ± 1.7	6.6 ± 1.2	9.0 ± 1.6	8.5 ± 1.2
**3**	34.8 ± 2.3	55.2 ± 2.6	22.7 ± 2.8	57.1 ± 2.5
**4**	42.3 ± 3.7	57.5 ± 2.4	65.4 ± 3.1	59.7 ± 1.9
**5**	5.9 ± 1.1	11.1 ± 1.8	7.9 ± 2.0	5.3 ± 2.1
**6**	9.6 ± 2.3	7.8 ± 1.1	8.4 ± 1.2	8.8 ± 1.1
**7**	22.5 ± 1.9	24.8 ± 2.6	57.5 ± 2.9	30.6 ± 2.8
**8**	27.6 ± 2.9	16.6 ± 1.9	13.4 ± 2.1	26.2 ± 1.8
**9**	47.8 ± 3.2	24.8 ± 6.2	45.6 ± 2.8	52.4 ± 3.2
carbendazim ^a^	80.2 ± 2.2	91.0 ± 3.2	87.2 ± 3.2	86.6 ± 2.1

^a^ Carbendazim was used as a positive control. Values are shown as mean ± standard deviation (n = 3).

## Data Availability

The original contributions presented in the study are included in the article/supplementary material, further inquiries can be directed to the corresponding authors.

## Supplementary Materials

The following supporting information can be downloaded at: https://www.mdpi.com/article/10.3390/jof11040275/s1, Figure S1: ^1^H NMR (600 MHz, CD_3_OD) spectrum of **1**; Figure S2: ^13^C NMR (150 MHz, CD_3_OD) spectrum of **1**; Figure S3: HSQC (600 MHz, CD_3_OD) spectrum of **1**; Figure S4: HMBC (600 MHz, CD_3_OD) spectrum of **1**; Figure S5: ^1^H-^1^H COSY (600 MHz, CD_3_OD) spectrum of **1**. Figure S6: NOESY spectrum of **1**; Figure S7: HRESIMS spectrum of **1**. Figure S8: IR spectrum of **1**; Figure S9: The UV spectrum of **1**. Figure S10: ^1^H NMR (600 MHz, CD_3_OD) spectrum of **2**; Figure S11: ^13^C NMR (150 MHz, CD_3_OD) spectrum of **2**; Figure S12: HSQC (600 MHz, CD_3_OD) spectrum of **2**; Figure S13: HMBC (600 MHz, CD_3_OD) spectrum of **2**; Figure S14: ^1^H-^1^H COSY (600 MHz, CD_3_OD) spectrum of **2**; Figure S15: NOESY spectrum of **2**; Figure S16: HRESIMS spectrum of **2**; Figure S17: IR spectrum of **2**; Figure S18: The UV spectrum of compound **2**; Figure S19: ^1^H NMR (600 MHz, CD_3_OD) spectrum of **3**; Figure S20: ^13^C NMR (150 MHz, CD_3_OD) spectrum of **3**; Figure S21: HSQC (600 MHz, CD_3_OD) spectrum of **3**; Figure S22: HMBC (600 MHz, CD_3_OD) spectrum of **3**; Figure S23: ^1^H-^1^H COSY (600 MHz, CD_3_OD) spectrum of **3**; Figure S24: HRESIMS spectrum of **3**; Figure S25: IR spectrum of **3**; Figure S26: The UV spectrum of compound **3**; Figure S27: ^1^H NMR (600 MHz, CD_3_OD) spectrum of **4**; Figure S28: ^13^C NMR (150 MHz, CD_3_OD) spectrum of **4**; Figure S29: HSQC (600 MHz, CD_3_OD) spectrum of **4**; Figure S30: HMBC (600 MHz, CD_3_OD) spectrum of **4**; Figure S31: ^1^H-^1^H COSY (600 MHz, CD_3_OD) spectrum of **4**; Figure S32: HRESIMS spectrum of **4**; Figure S33: IR spectrum of **4**; Figure S34: The UV spectrum of compound **4**.
