# Supplementary material for: New Bioactive Sesquiterpeniods from the Plant-Derived Endophytic Fungus Schizophyllum sp. HM230
Source: J Fungi (Basel). 2025 Apr 1;11(4):275. doi: 10.3390/jof11040275 (PMC12029045; doi:10.3390/jof11040275)
Supplement: Supplementary file 1 [file jof-11-00275-s001.zip › jof-3539498-supplementary.pdf]

New Bioactive Sesquiterpeniods from the Plant-derived Endophytic Fungus *Schizophyllum* sp. HM230

Shi-Yu Li[1], Lan Yao[2], Jian-Hua Lv[3], Zhuang Li[3], Shuai Xu[1], Yu Li[1,4], Dan Li[1,4,*] and Chang-Tian Li[1,4,*]

[1] Engineering Research Center of Chinese Ministry of Education for Edible and Medicinal Fungi, Jilin Agricultural University, Changchun 130118, China; lishiyu8866@126.com (S.-Y.L.); xushuai@jlau.edu.cn (S.X.); yuli966@126.com (Y.L.)

[2] Institute of Biology, Hebei Academy of Science, Shijiazhuang 050000, China; yl52wy@126.com

[3]College of Life Sciences, Hebei Normal University, Shijiazhuang 050000, China; lvjianhua@hebtu.edu.cn (J.-H.L.); lizhuang@hebtu.edu.cn (Z.L.)

[4]National-Local Joint Engineering Research Center of Economic Fungus, Jilin Agricultural University, Changchun 130118, China

* Correspondence: lidan@jlau.edu.cn(D.L.); lct@jlau.edu.cn (C.-T.L.)

Table of contents

Figure S1. ^{1}H NMR (600 MHz, CD_3OD) spectrum of 1

Figure S2. ^{13}C NMR (150 MHz, CD_3OD) spectrum of 1

Figure S3. HSQC (600 MHz, CD_3OD) spectrum of 1

Figure S4. HMBC (600 MHz, CD_3OD) spectrum of 1

Figure S5. ^{1}H-^{1}H COSY (600 MHz, CD_3OD) spectrum of 1

Figure S6. NOESY spectrum of 1

Figure S7. HRESIMS spectrum of 1

Figure S8. IR spectrum of 1

Figure S9. The UV spectrum of compound 1

Figure S10. ^{1}H NMR (600 MHz, CD_3OD) spectrum of 2

Figure S11. ^{13}C NMR (150 MHz, CD_3OD) spectrum of 2

Figure S12. HSQC (600 MHz, CD_3OD) spectrum of 2

Figure S13. HMBC (600 MHz, CD_3OD) spectrum of 2

Figure S14. ^{1}H-^{1}H COSY (600 MHz, CD_3OD) spectrum of 2

Figure S15. NOESY spectrum of 2

Figure S16. HRESIMS spectrum of 2

Figure S17. IR spectrum of 2

Figure S18. The UV spectrum of compound 2

Figure S19. ^{1}H NMR (600 MHz, CD_3OD) spectrum of 3

Figure S20. ^{13}C NMR (150 MHz, CD_3OD) spectrum of 3

Figure S21. HSQC (600 MHz, CD_3OD) spectrum of 3

Figure S22. HMBC (600 MHz, CD_3OD) spectrum of 3

Figure S23. ^{1}H-^{1}H COSY (600 MHz, CD_3OD) spectrum of 3

Figure S24. HRESIMS spectrum of 3

Figure S25. IR spectrum of 3

Figure S26. The UV spectrum of compound 3

Figure S27. ^{1}H NMR (600 MHz, CD_3OD) spectrum of 4

Figure S28. ^{13}C NMR (150 MHz, CD_3OD) spectrum of 4

Figure S29. HSQC (600 MHz, CD_3OD) spectrum of 4

Figure S30. HMBC (600 MHz, CD_3OD) spectrum of 4

Figure S31. ^1H-^1H COSY (600 MHz, CD_3OD) spectrum of 4

Figure S32. HRESIMS spectrum of 4

Figure S33. IR spectrum of 4

Figure S34. The UV spectrum of compound 4

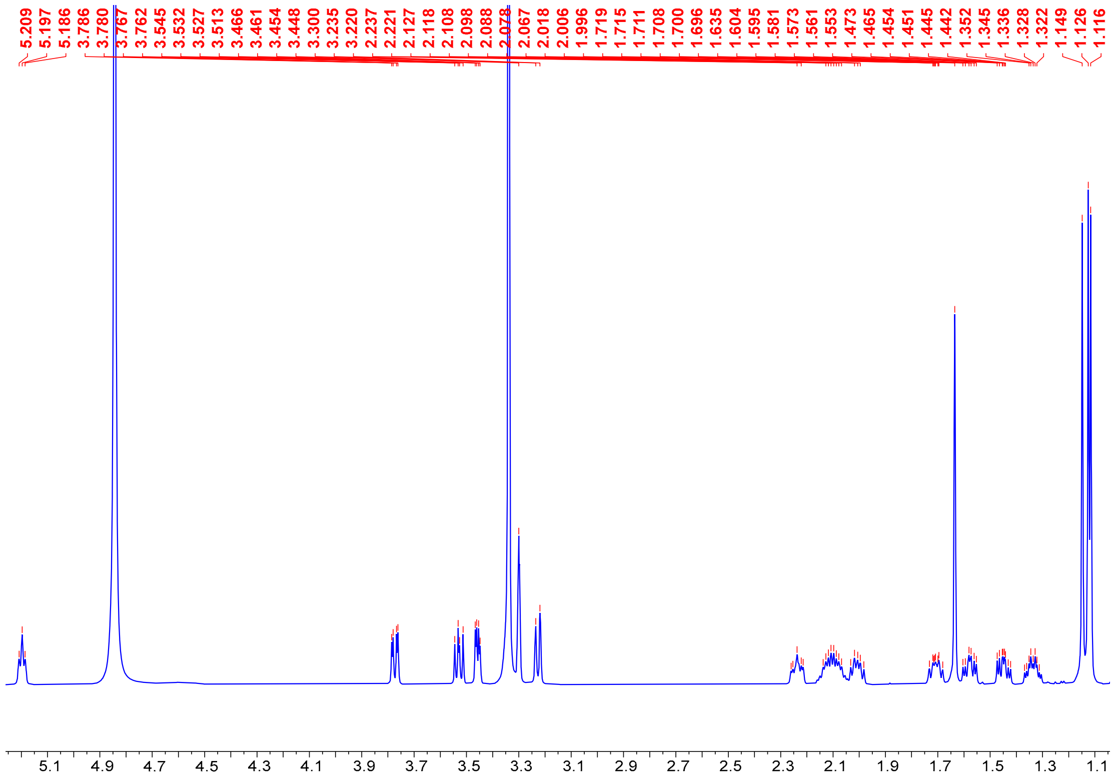

Figure S1. ^{1}H NMR (600 MHz, CD_3OD) spectrum of 1

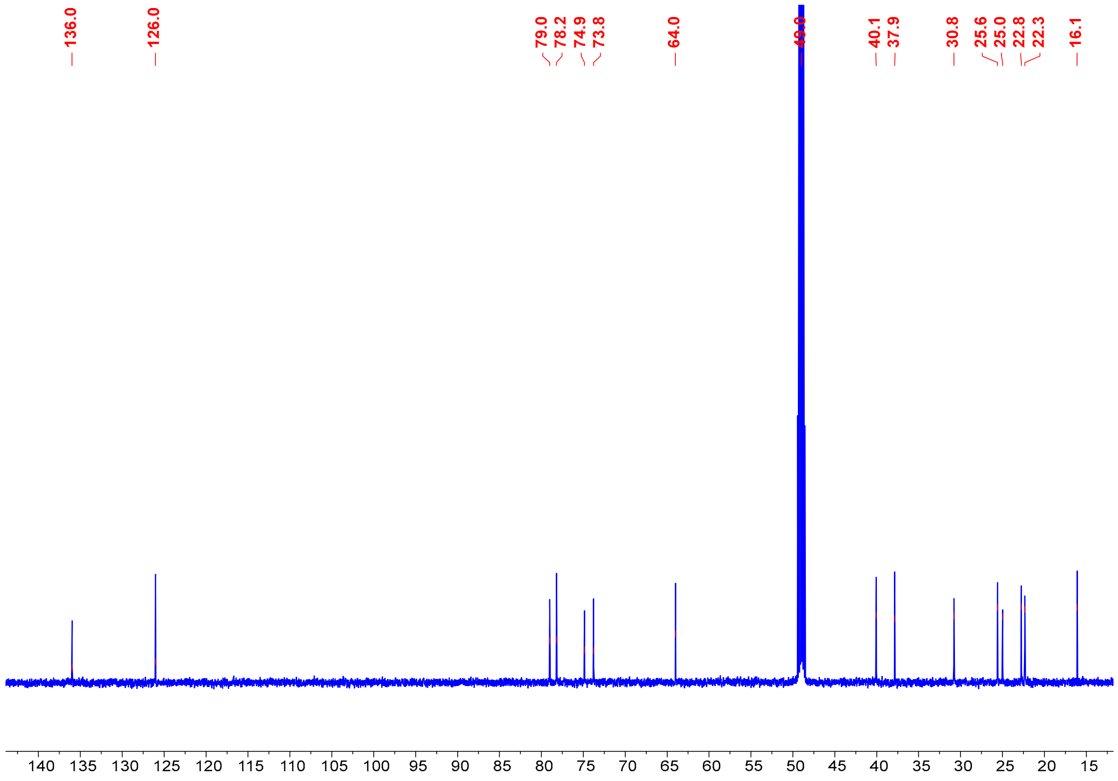

Figure S2. ^{13}C NMR (150 MHz, CD_3OD) spectrum of 1

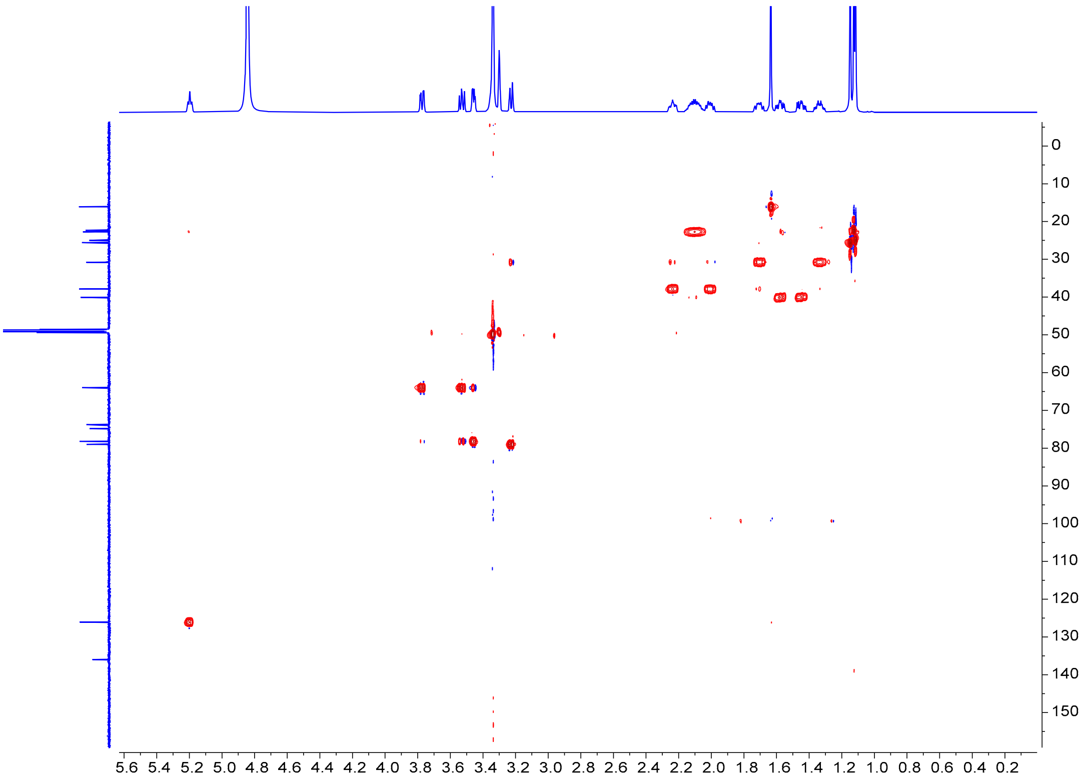

Figure S3. HSQC (600 MHz, CD3OD) spectrum of 1

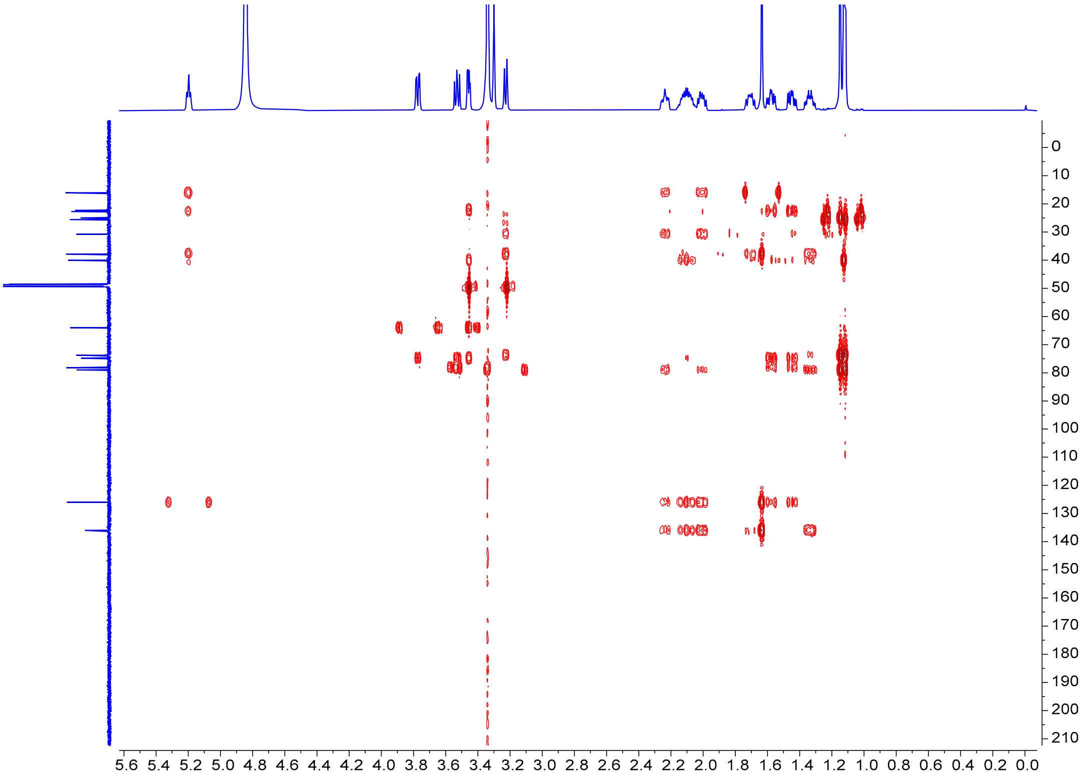

Figure S4. HMBC (600 MHz, CD3OD) spectrum of 1

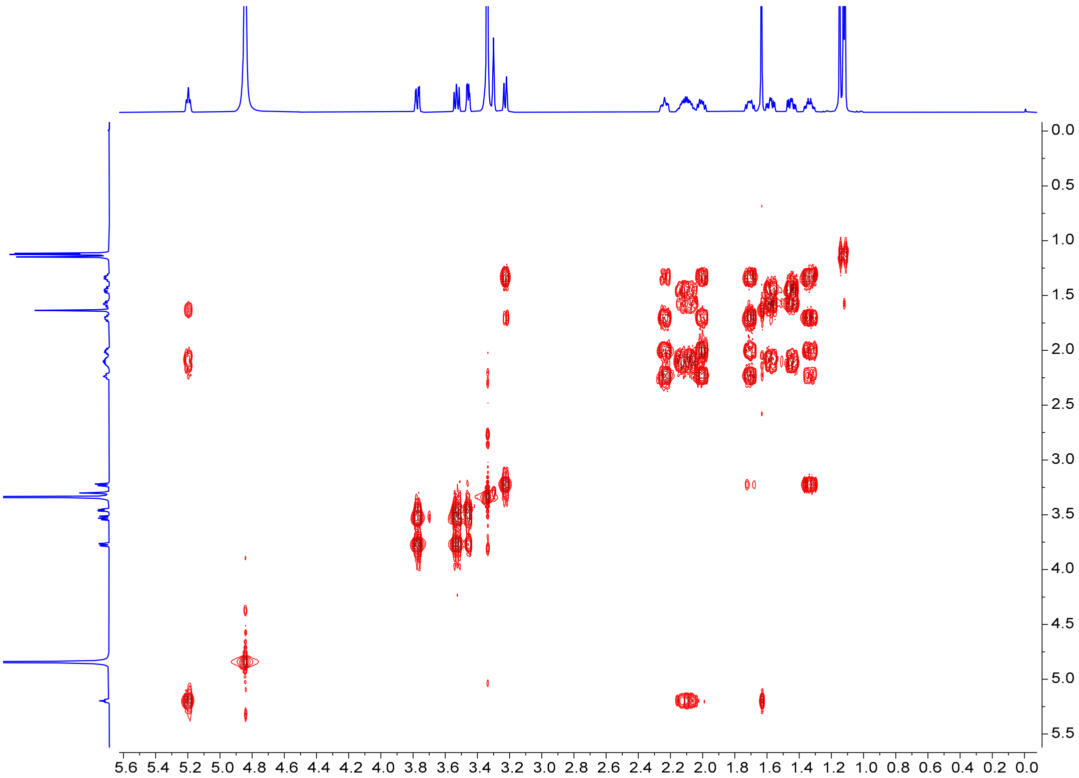

Figure S5. ^{1}H-^{1}H COSY (600 MHz, CD_3OD) spectrum of 1

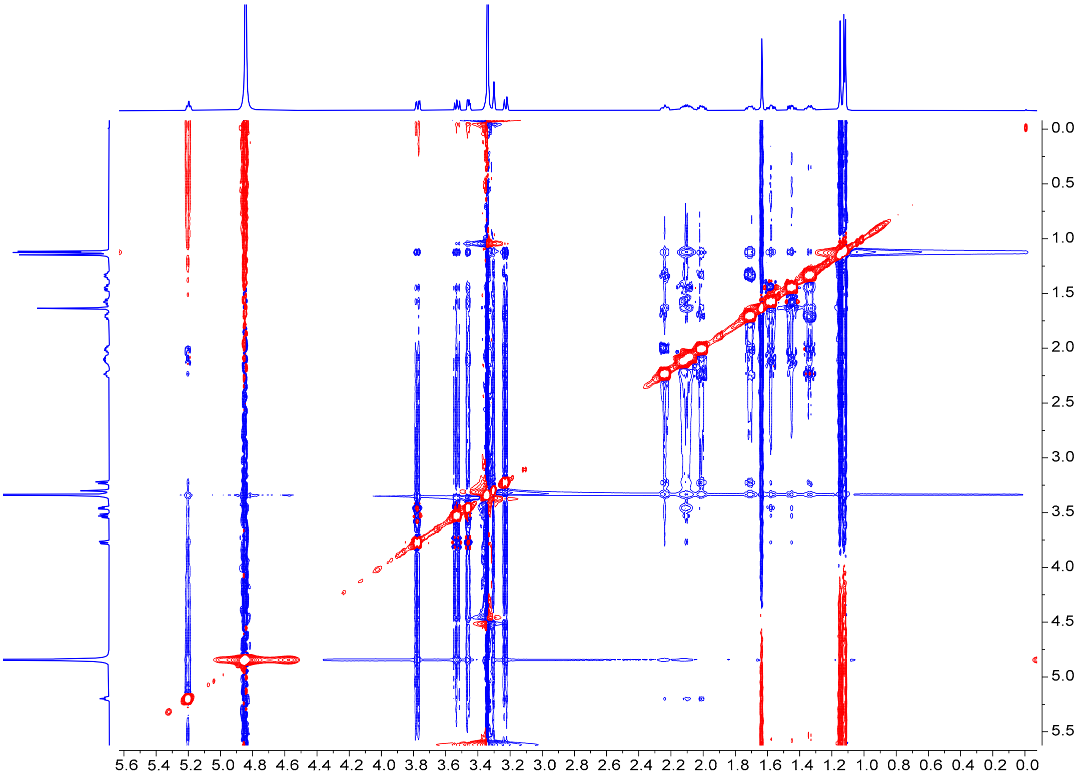

Figure S6. NOESY spectrum of 1

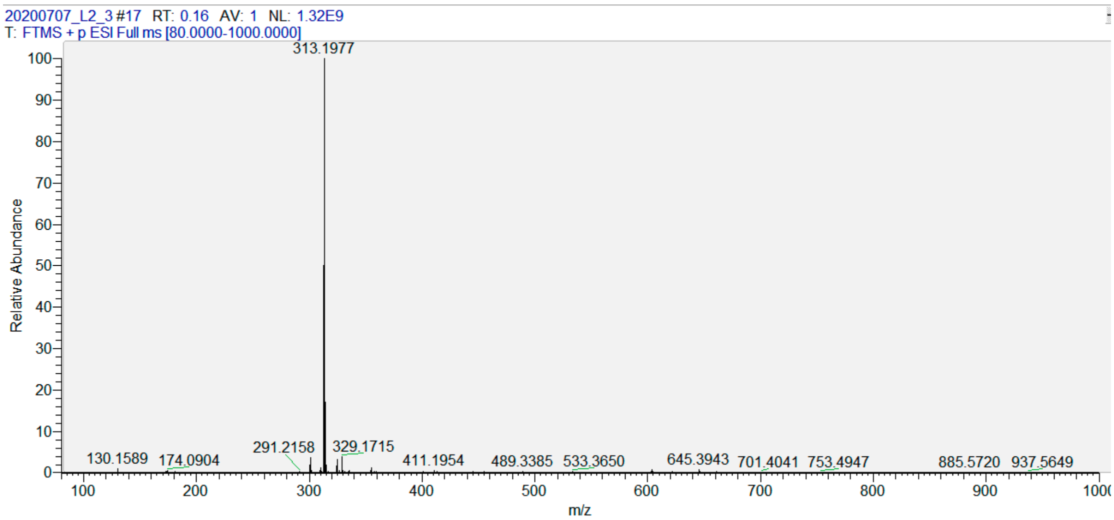

Figure S7. HRESIMS spectrum of 1

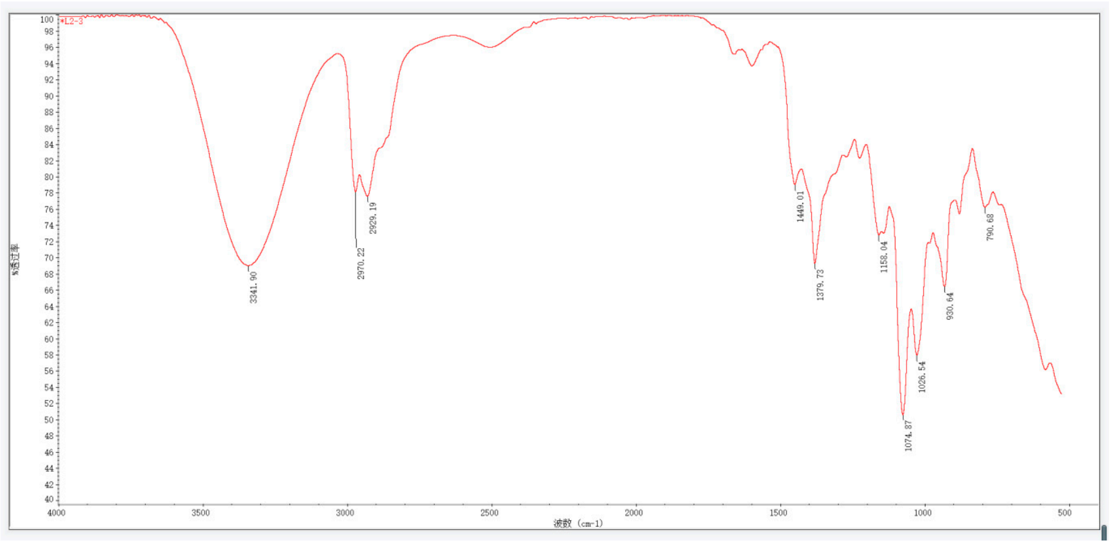

Figure S8. IR spectrum of 1

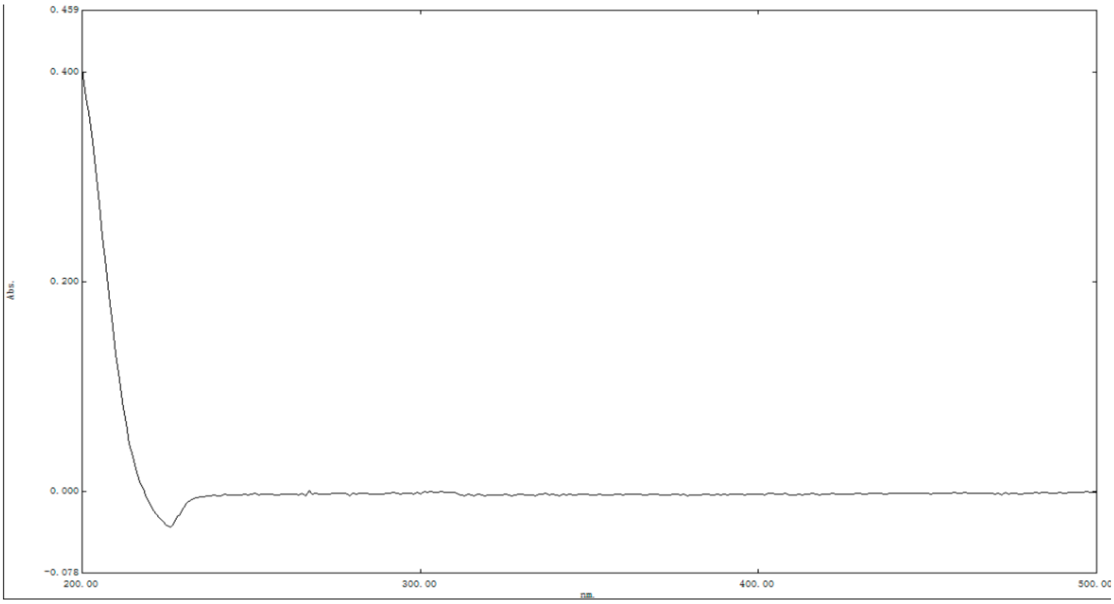

Figure S9. The UV spectrum of compound 1

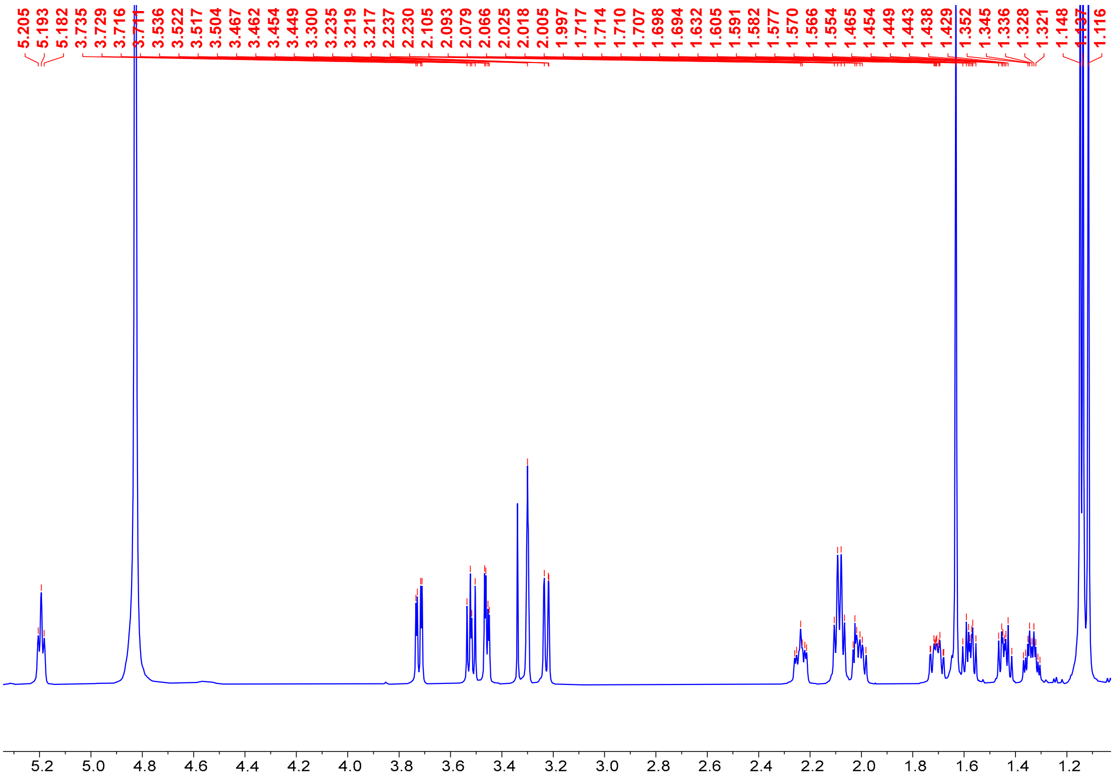

Figure S10. ^{1}H NMR (600 MHz, CD_3OD) spectrum of 2

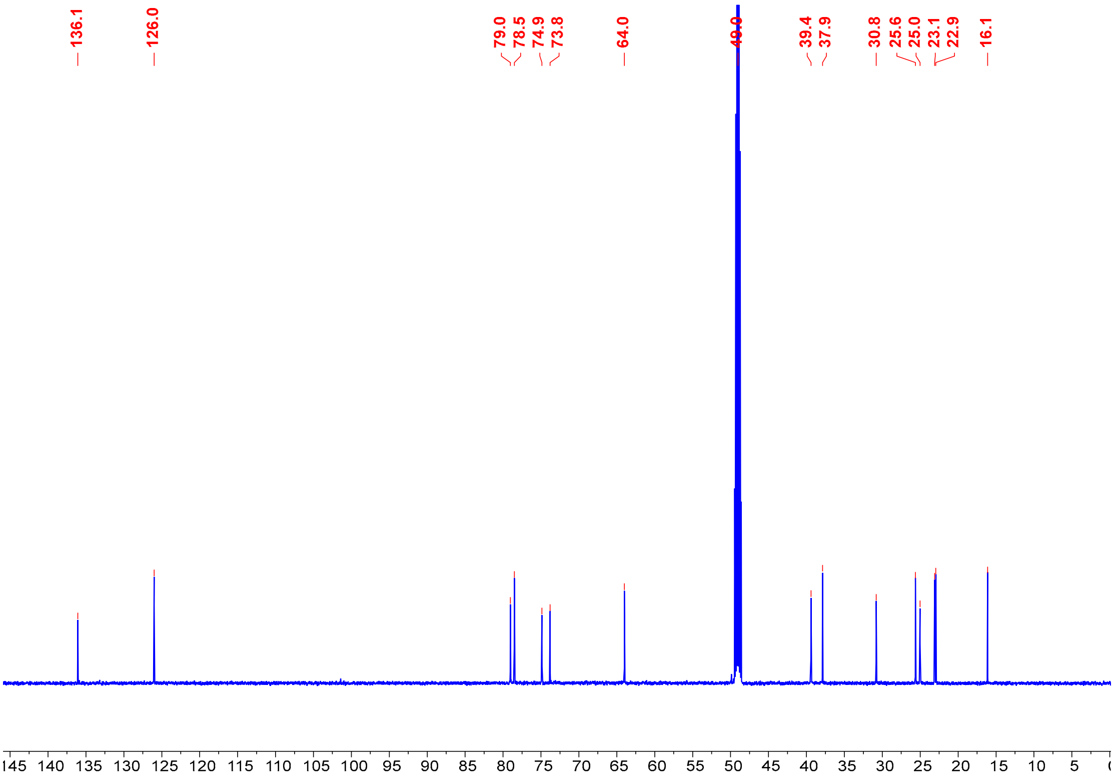

Figure S11. ^{13}C NMR (150 MHz, CD_3OD) spectrum of 2

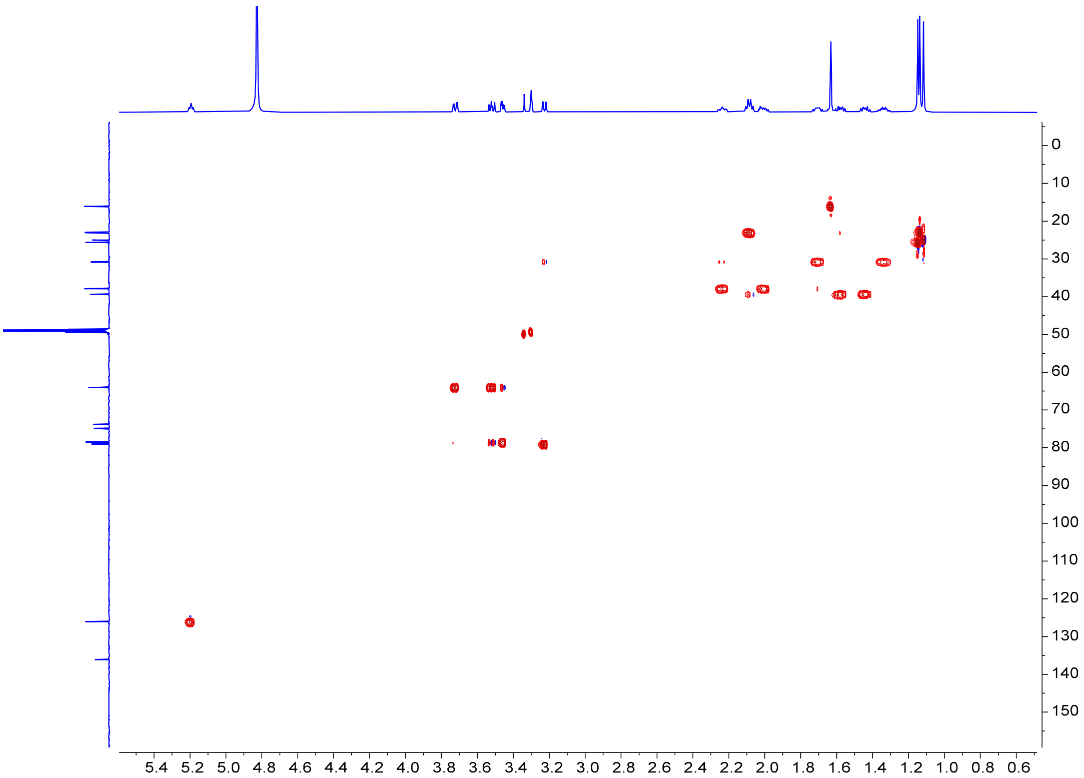

Figure S12. HSQC (600 MHz, CD3OD) spectrum of 2

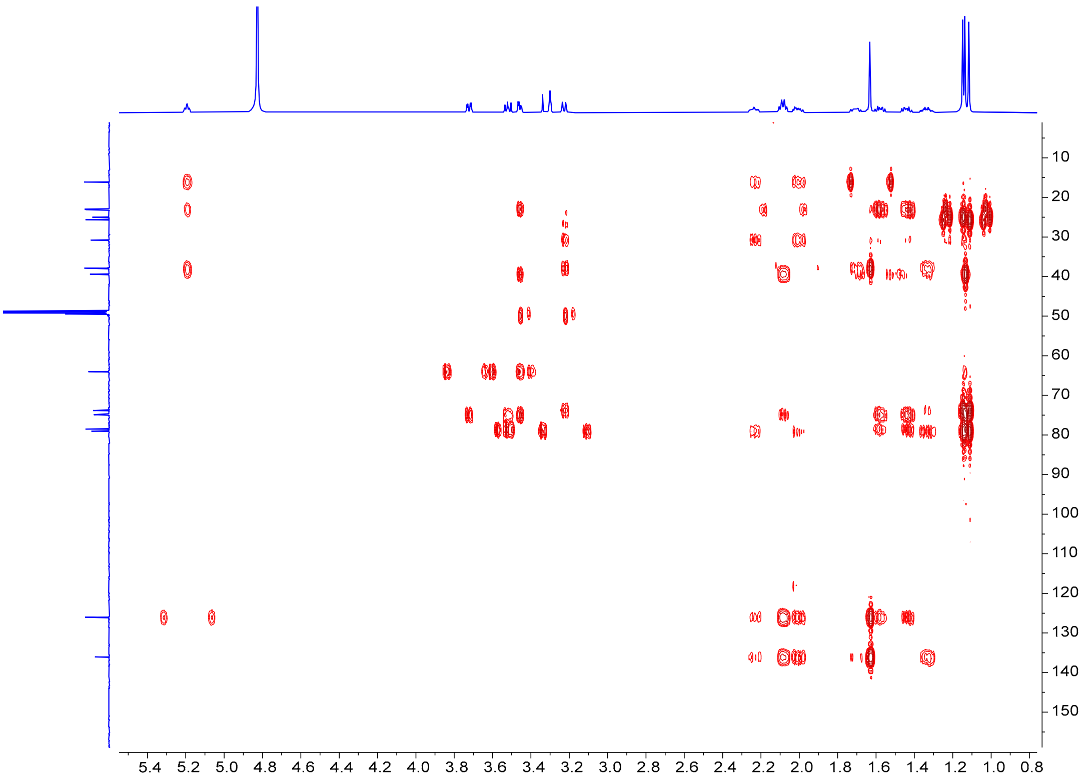

Figure S13. HMBC (600 MHz, CD3OD) spectrum of 2

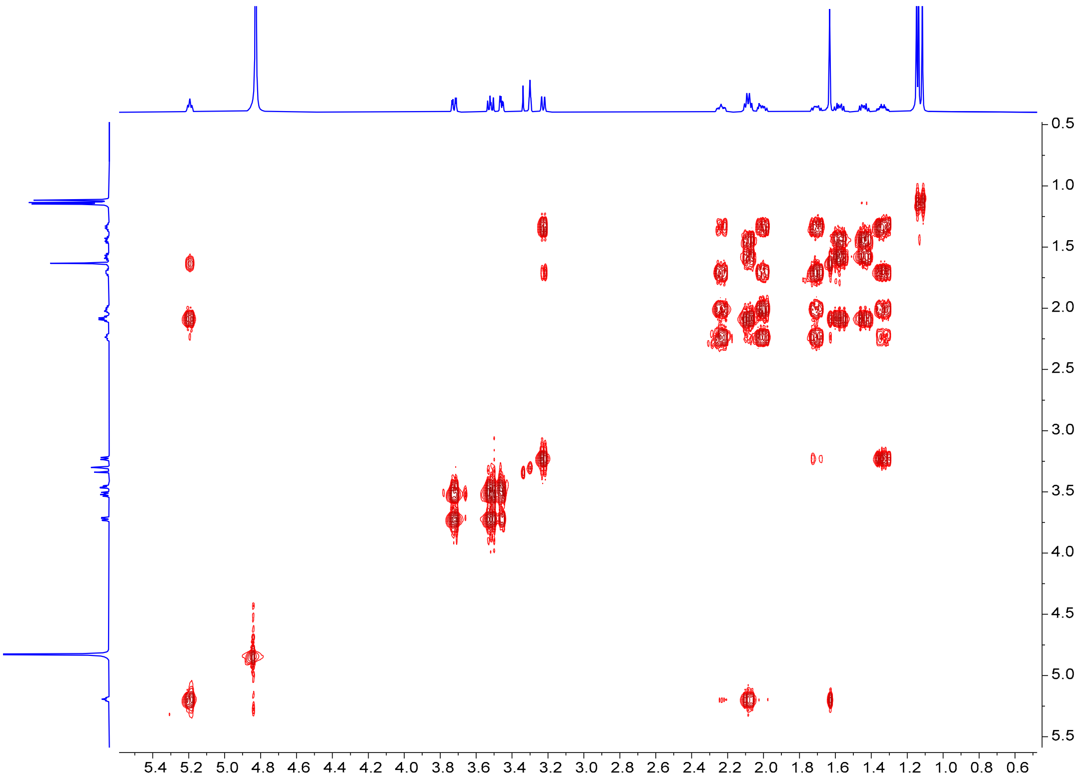

Figure S14. ^{1}H-^{1}H COSY (600 MHz, CD_3OD) spectrum of 2

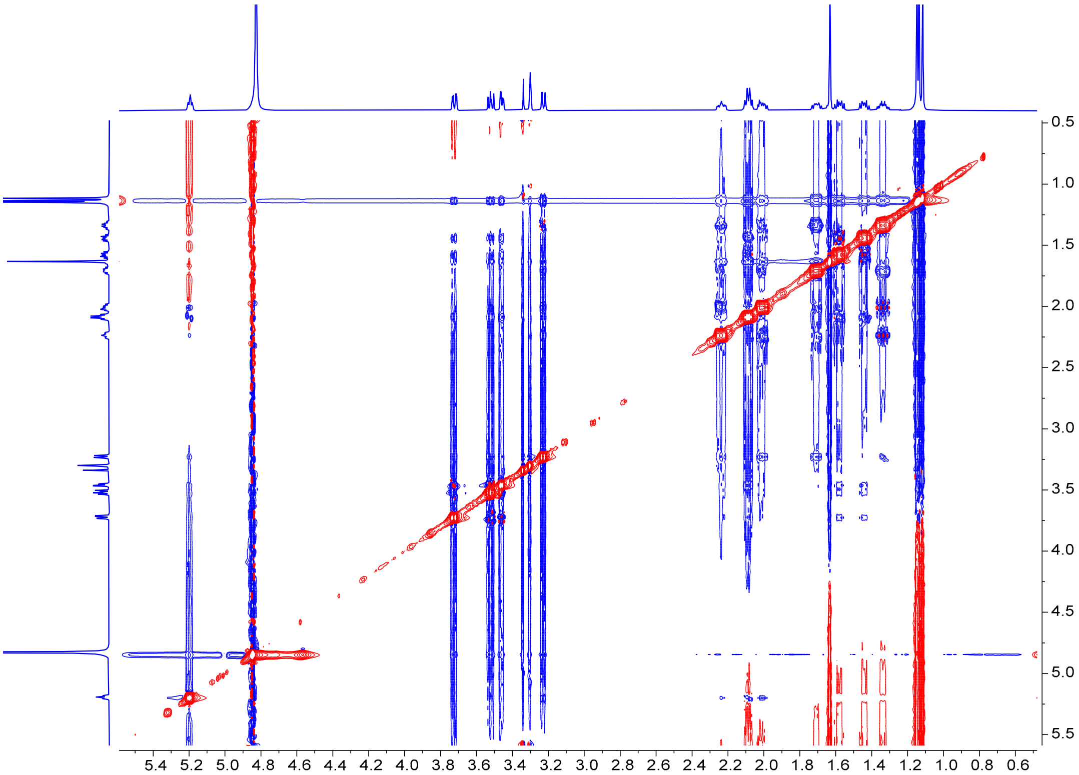

Figure S15. NOESY spectrum of 2

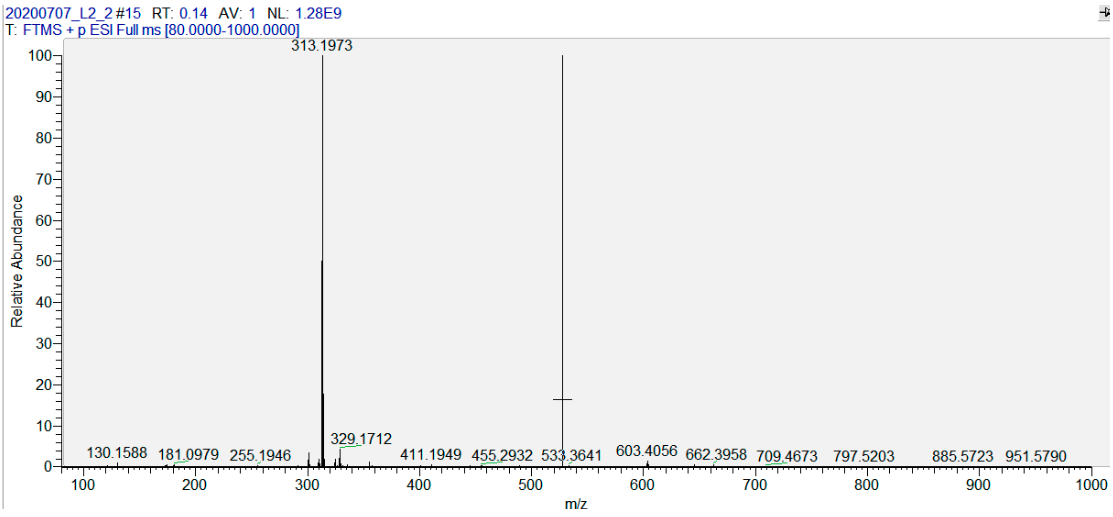

Figure S16. HRESIMS spectrum of 2

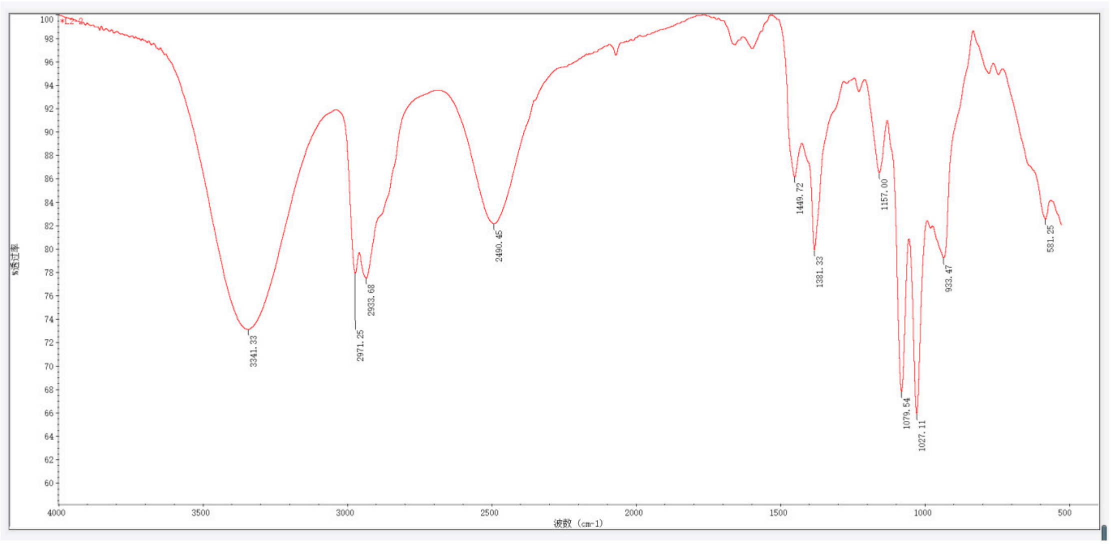

Figure S17. IR spectrum of 2

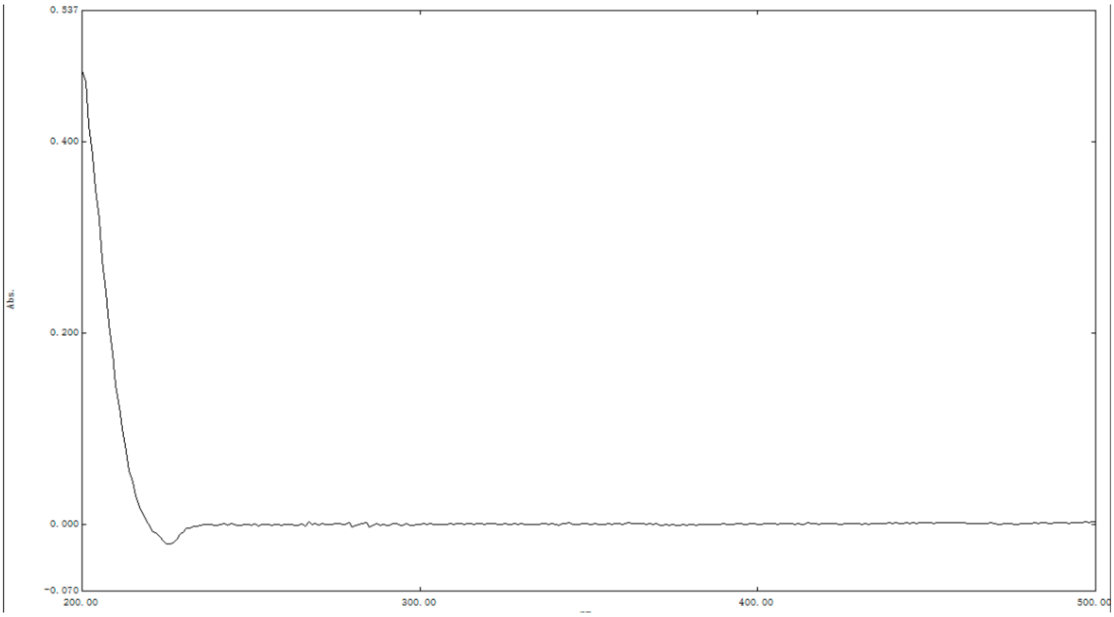

Figure S18. The UV spectrum of compound 2

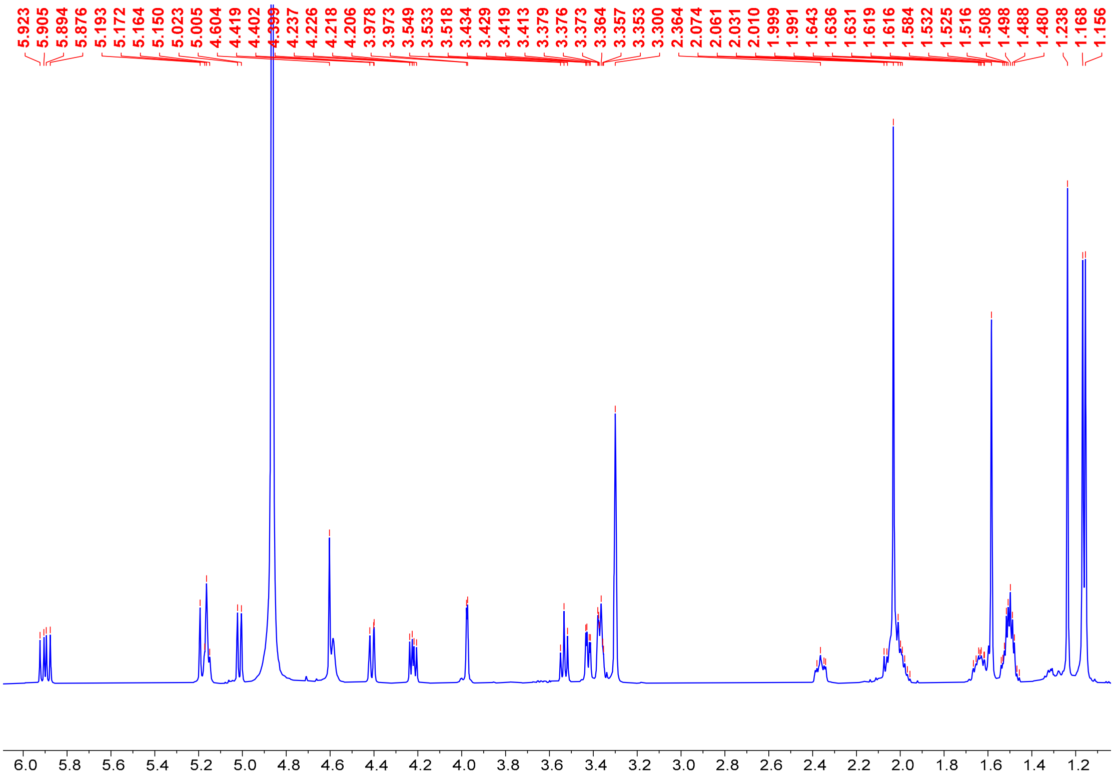

Figure S19. ^{1}H NMR (600 MHz, CD_3OD) spectrum of 3

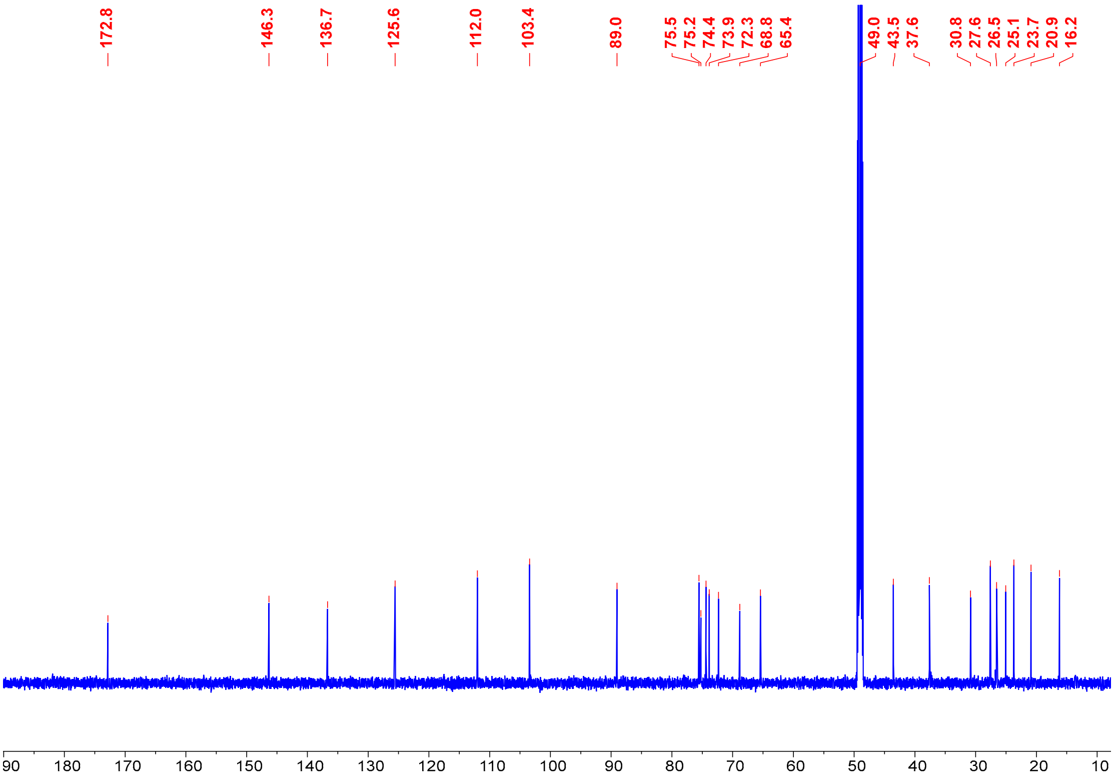

Figure S20. ^{13}C NMR (150 MHz, CD_3OD) spectrum of 3

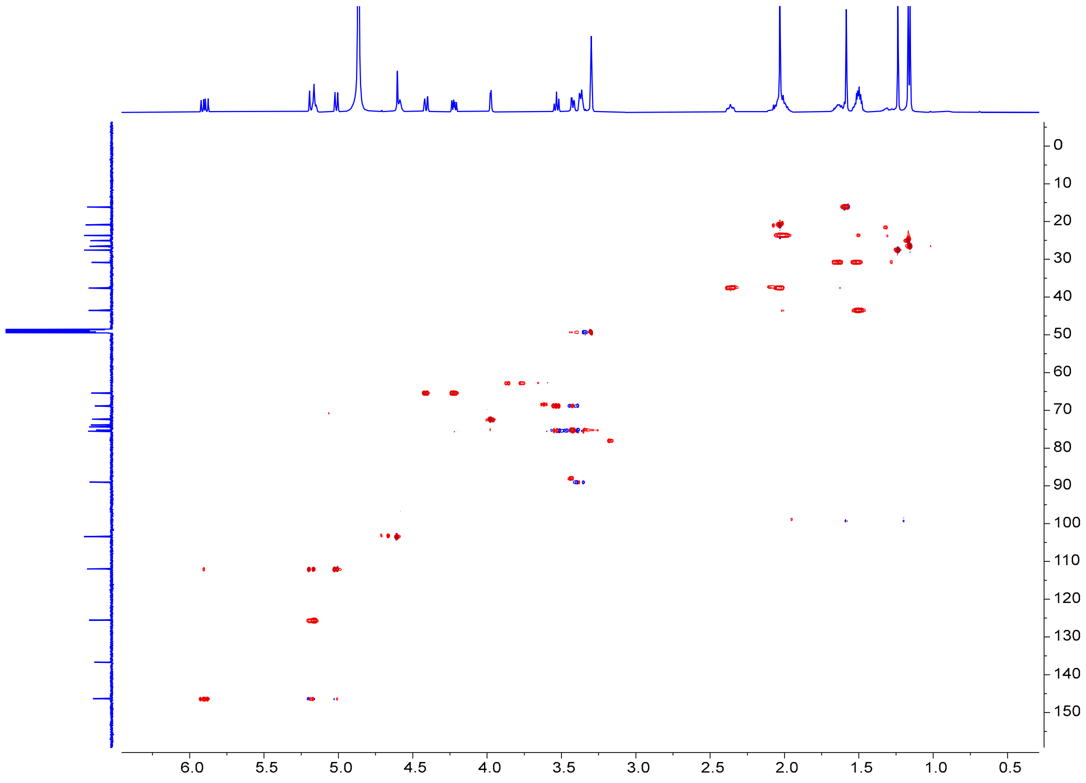

Figure S21. HSQC (600 MHz, CD3OD) spectrum of 3

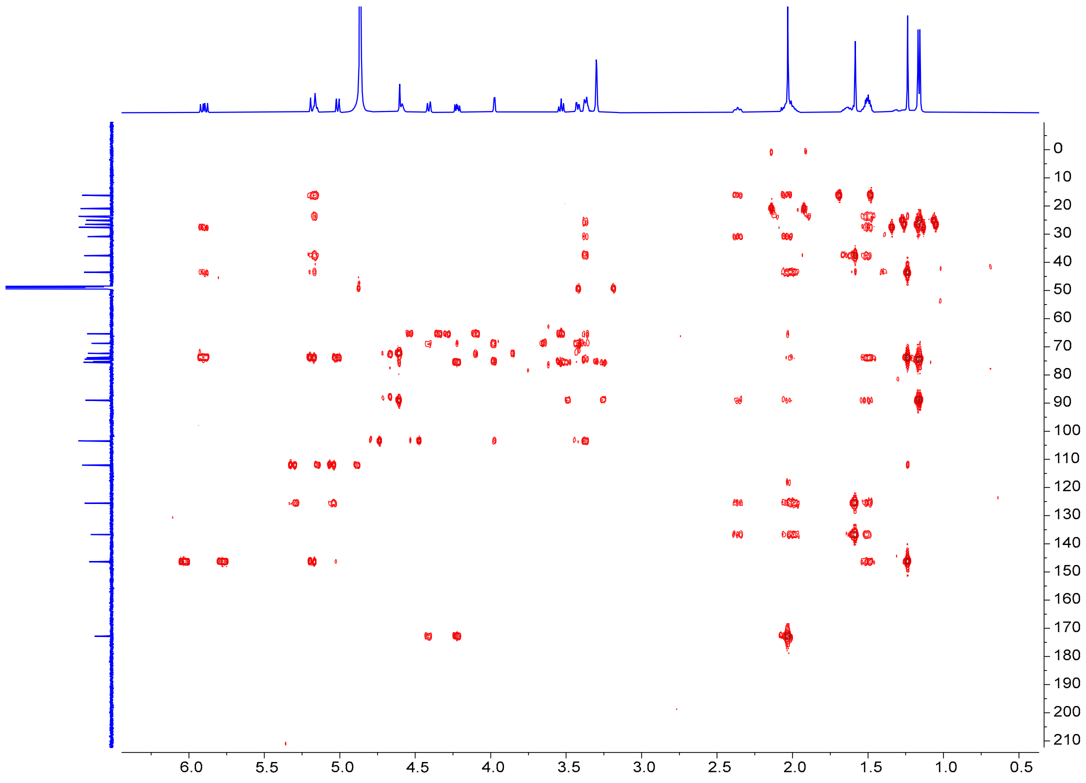

Figure S22. HMBC (600 MHz, CD3OD) spectrum of 3

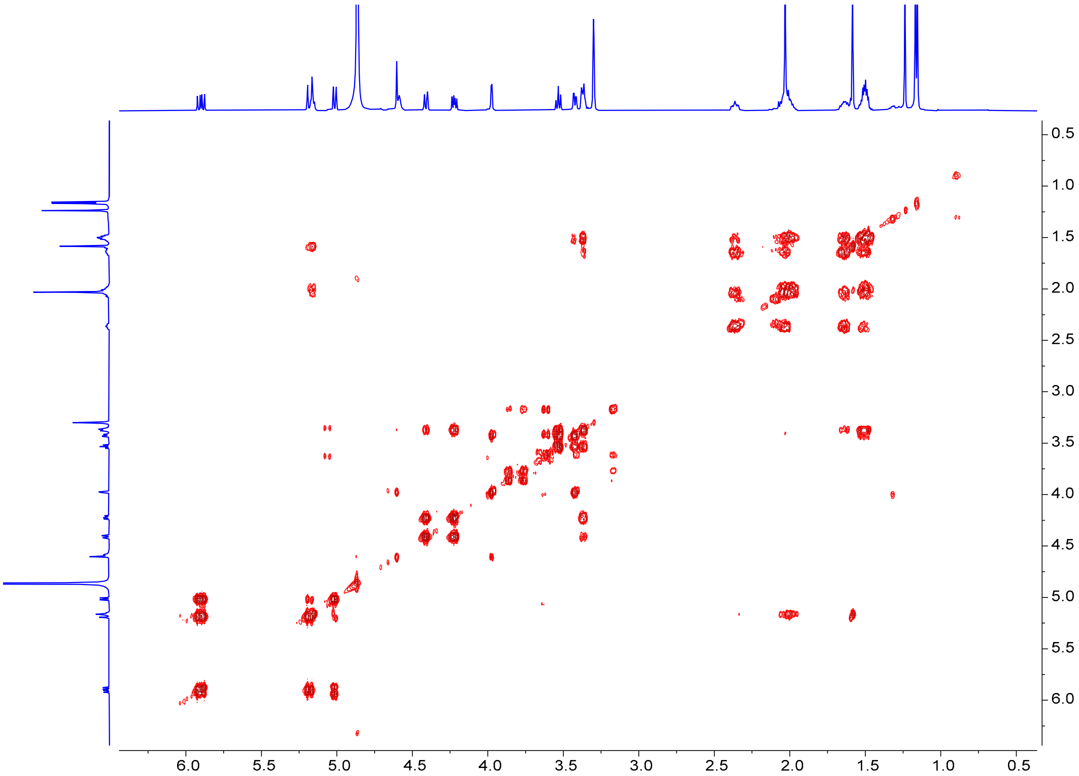

Figure S23. ^{1}H-^{1}H COSY (600 MHz, CD_3OD) spectrum of 3

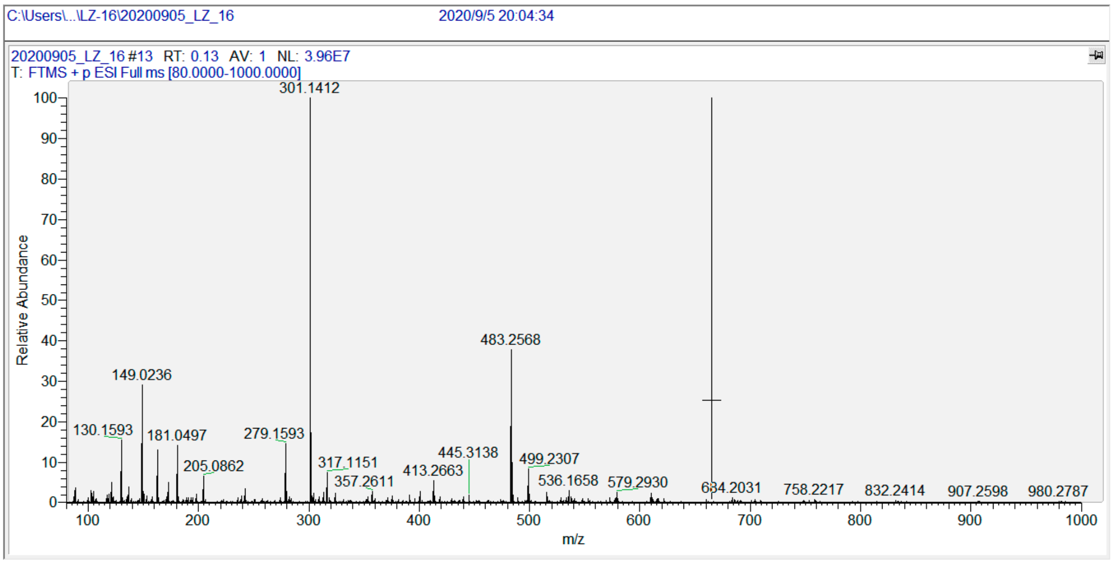

Figure S24. HRESIMS spectrum of 3

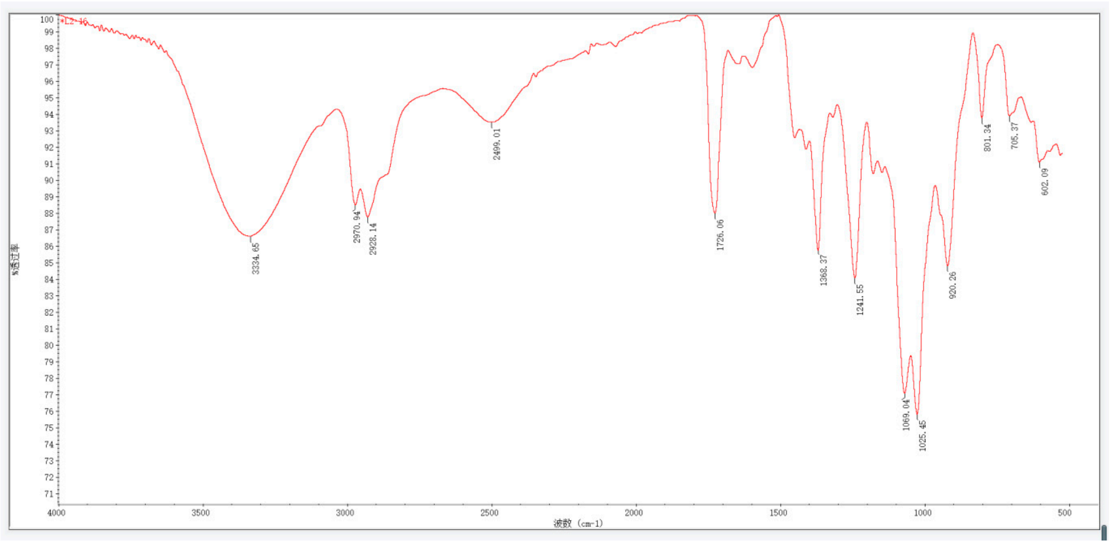

Figure S25. IR spectrum of 3

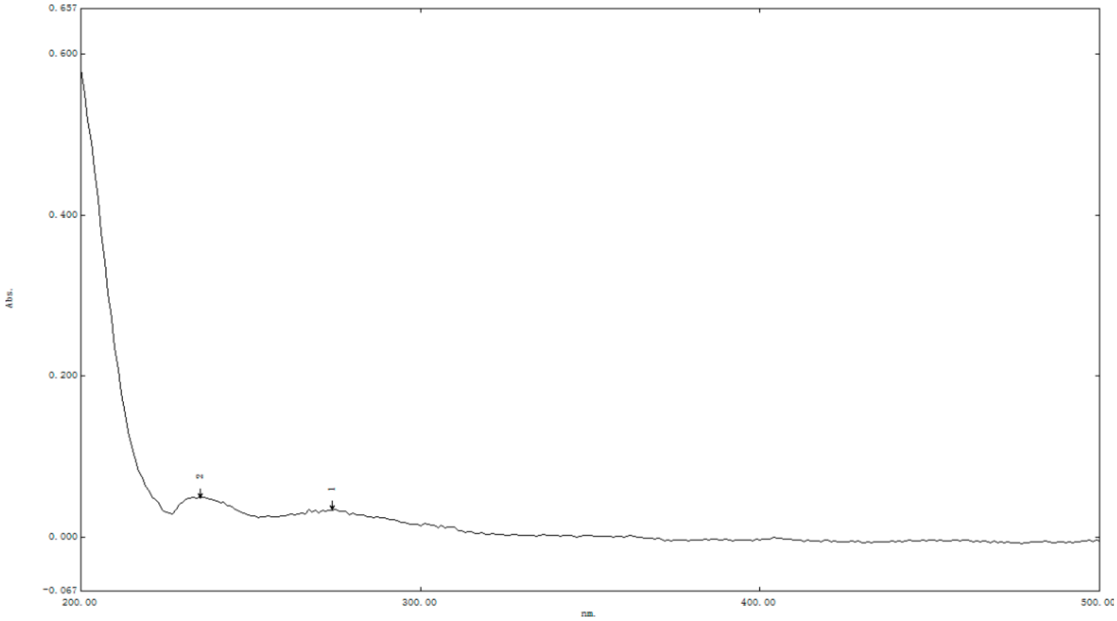

Figure S26. The UV spectrum of compound 3

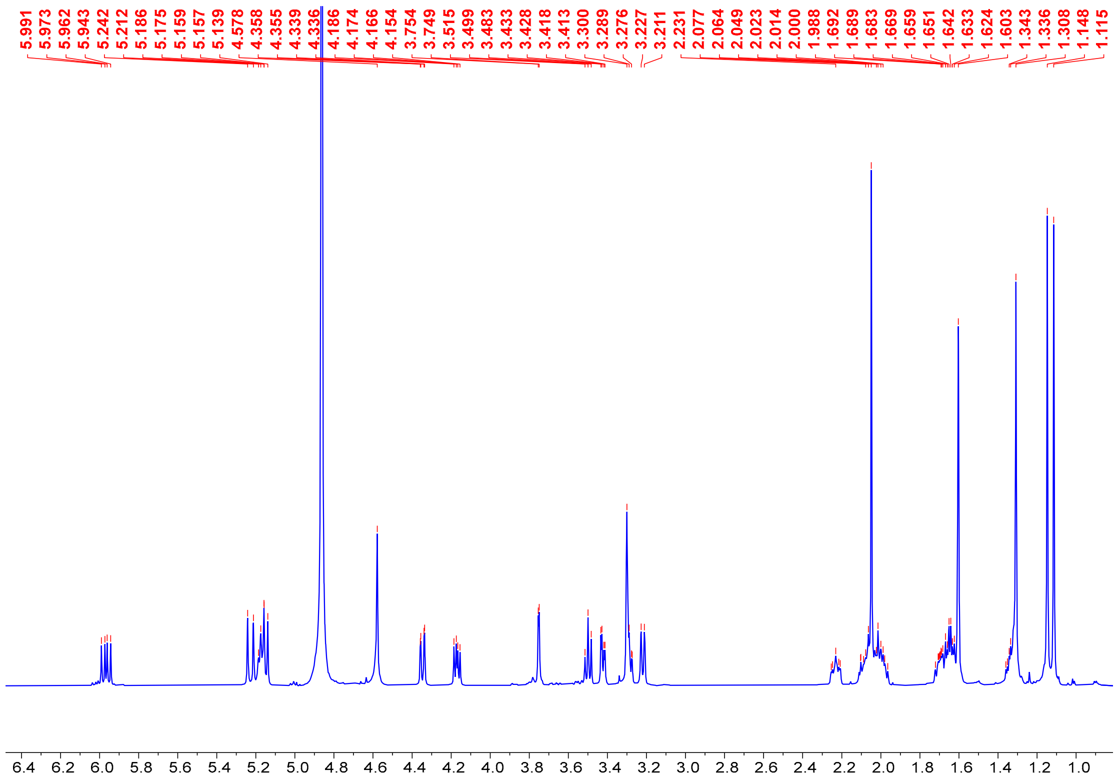

Figure S27. ^{1}H NMR (600 MHz, CD_3OD) spectrum of 4

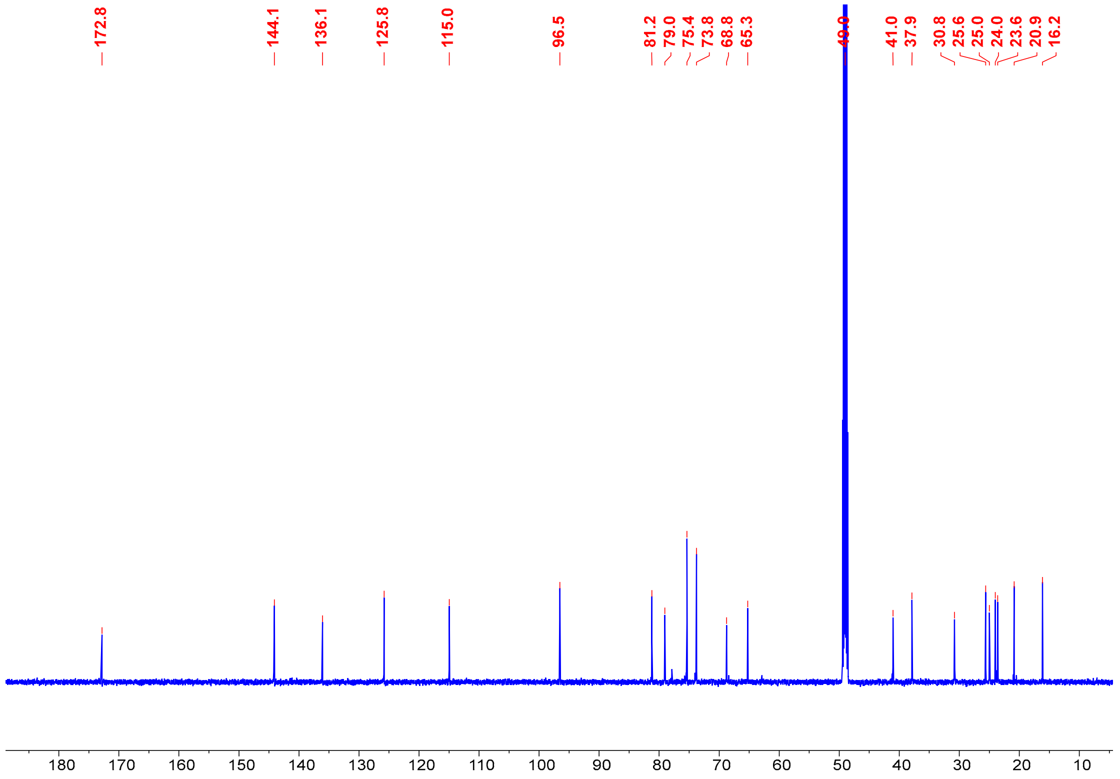

Figure S28. ^{13}C NMR (150 MHz, CD_3OD) spectrum of 4

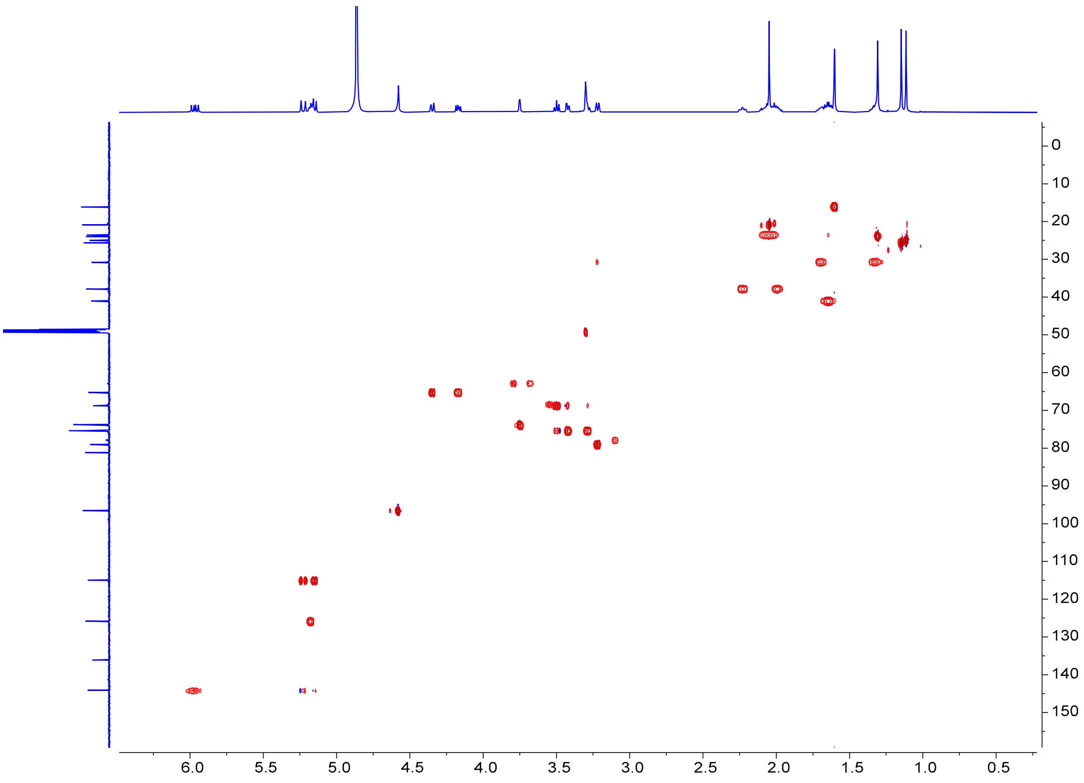

Figure S29. HSQC (600 MHz, CD_3OD) spectrum of 4

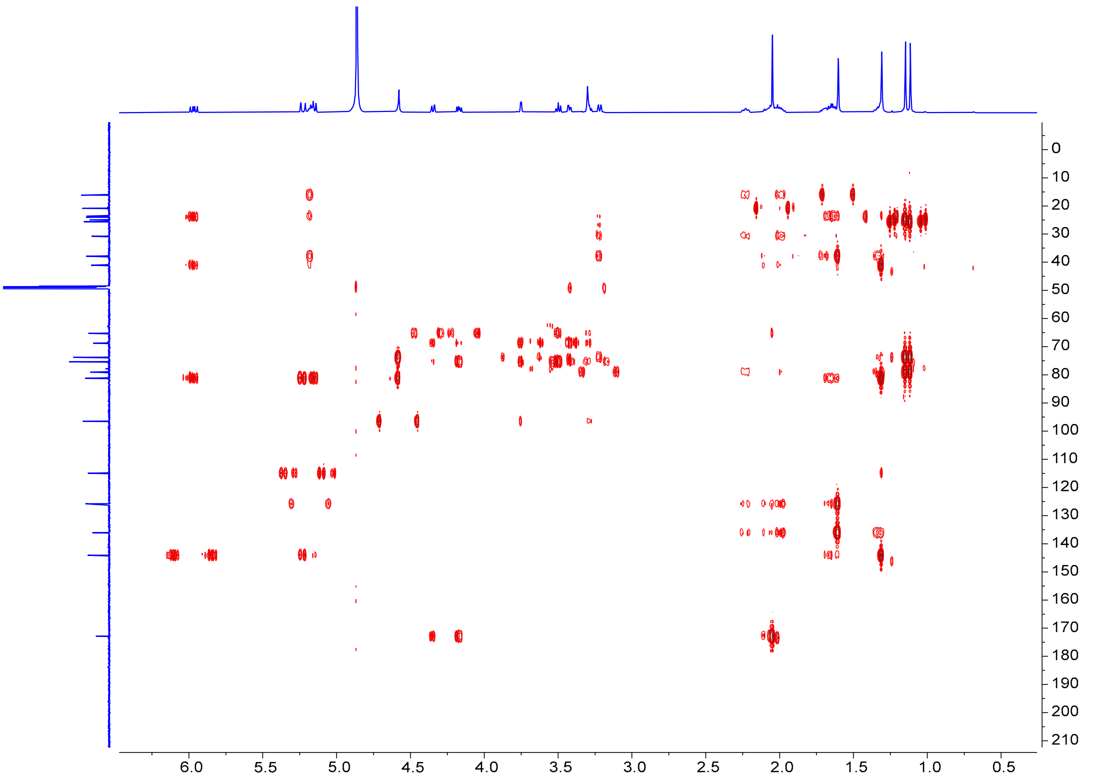

Figure S30. HMBC (600 MHz, CD_3OD) spectrum of 4

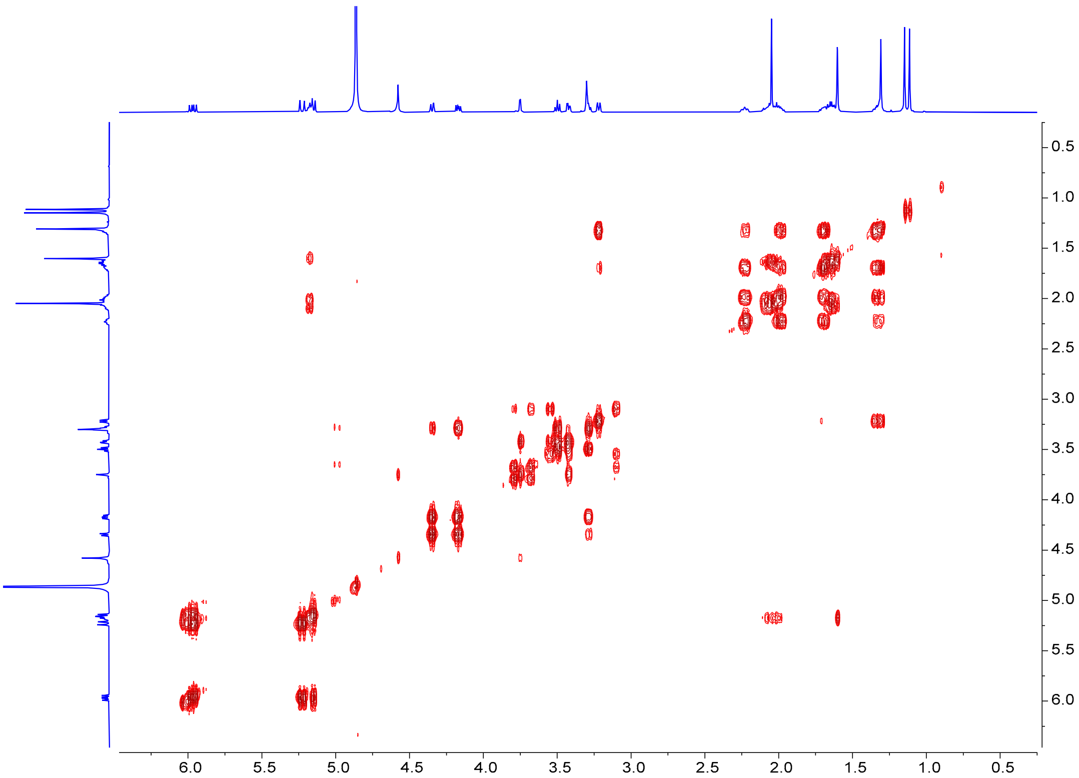

Figure S31. ^{1}H-^{1}H COSY (600 MHz, CD_3OD) spectrum of 4

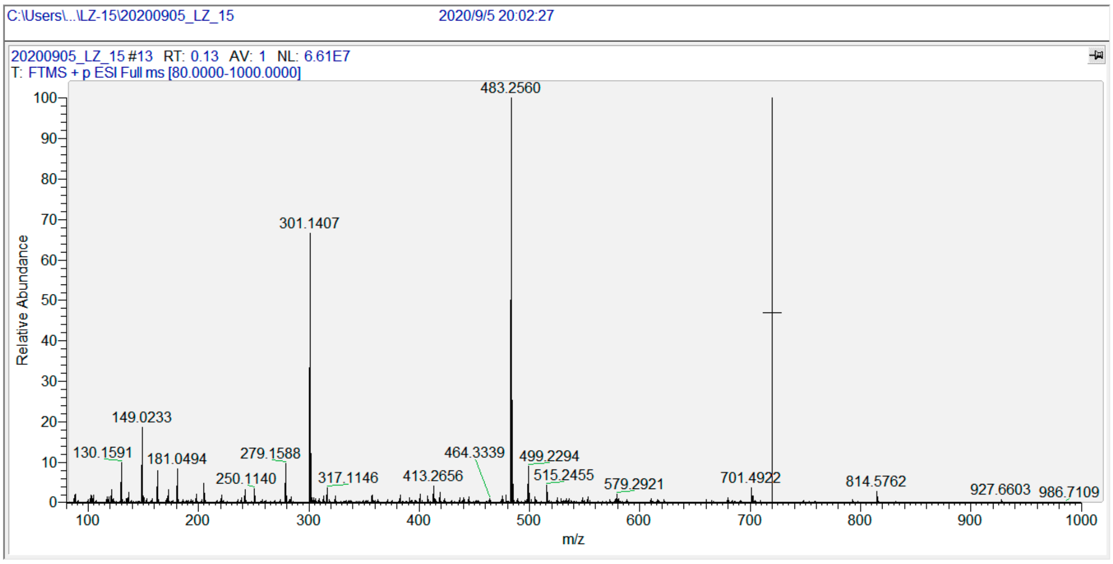

Figure S32. HRESIMS spectrum of 4

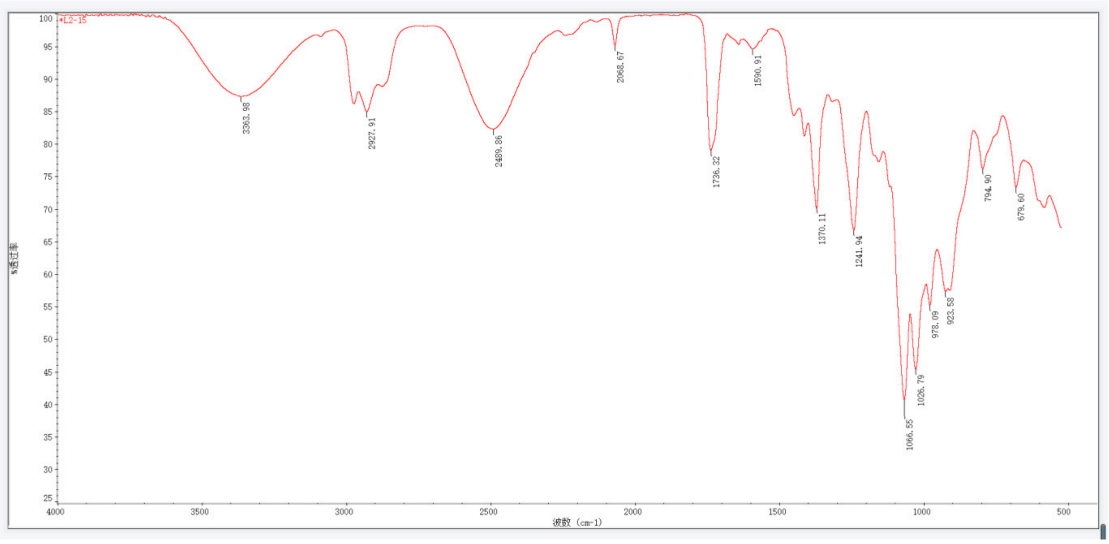

Figure S33. IR spectrum of 4

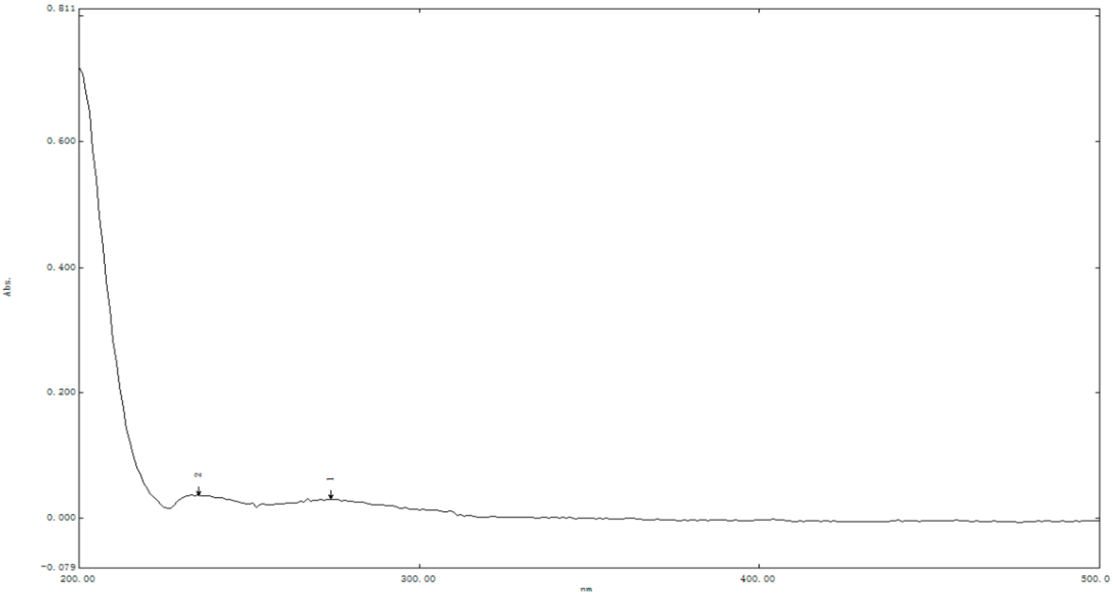

Figure S34. The UV spectrum of compound 4
